# Stepwise onset of the Icehouse world and its impact on Oligo-Miocene Central Asian mammals

**DOI:** 10.1038/srep36169

**Published:** 2016-11-29

**Authors:** Mathias Harzhauser, Gudrun Daxner-Höck, Paloma López-Guerrero, Olivier Maridet, Adriana Oliver, Werner E. Piller, Sylvain Richoz, Margarita A. Erbajeva, Thomas A. Neubauer, Ursula B. Göhlich

**Affiliations:** 1 Natural History Museum Vienna, Burgring 7, 1010 Vienna, Austria; 2 Departamento de Paleontología, Facultad de Ciencias Geológicas, C/José Antonio Novais, 2, Universidad Complutense de Madrid, 28040 Madrid, Spain; 3 Jurassica Museum, Fontenais 21, 2900 Porrentruy, Switzerland; 4 Department of Geosciences, Earth Sciences, University of Fribourg, Chemin du Musée 6, Pérolles, 1700 Fribourg, Switzerland; 5 Paleobiology Department, Museo Nacional de Ciencias Naturales-CSIC, C/José Gutiérrez Abascal, 2, 28006 Madrid, Spain; 6 Institute of Earth Sciences, Graz University, Heinrichstraße 26, 8010 Graz, Austria; 7 Geological Institute, Siberian Branch, Russian Academy of Sciences, Ulan-Ude; Sahianova Str., 6a, 670047 Ulan-Ude, Russia

## Abstract

Central Asia is a key area to study the impact of Cenozoic climate cooling on continental ecosystems. One of the best places to search for rather continuous paleontological records is the Valley of Lakes in Mongolia with its outstandingly fossil-rich Oligocene and Miocene terrestrial sediments. Here, we investigate the response by mammal communities during the early stage of Earth’s icehouse climate in Central Asia. Based on statistical analyses of occurrence and abundance data of 18608 specimens representing 175 mammal species and geochemical (carbon isotopes) and geophysical (magnetic susceptibility) data we link shifts in diversities with major climatic variations. Our data document for the first time that the post-Eocene aridification of Central Asia happened in several steps, was interrupted by short episodes of increased precipitation, and was not a gradual process. We show that the timing of the major turnovers in Oligocene mammal communities is tightly linked with global climate events rather than slow tectonics processes. The most severe decline of up 48% of total diversity is related to aridification during the maximum of the Late Oligocene Warming at 25 Ma. Its magnitude was distinctly larger than the community turnover linked to the mid-Oligocene Glacial Maximum.

The Tertiary terrestrial deposits of the Valley of Lakes in Mongolia (Fig. 1) are outstanding concerning their rich fossil record and the vast outcrops (Fig. 2). This fact makes the area a main target to study the development of continental faunas of Central Asia. The best documented and most dramatic faunal turnover in Central Asia is the Eocene-Oligocene Transition (EOT), known as the “Mongolian Remodeling”^1^. In the marine records, the EOT is marked by a sudden increase in deep-sea benthic foraminiferal δ^18^O-values, indicating the first step towards the late Cenozoic icehouse state^2^,^3^. The “Big Chill”, as the EOT was coined by Miller *et al*.^4^, obviously increased aridification in Central Asia^5^,^6^,^7^, although this trend seems to have been initiated earlier already in late Eocene times by the retreat of the proto-Paratethys Sea^8^,^9^. In addition, the intensified uplift of the Tibetan plateau and of the Hangay range (Fig. 1) amplified aridification^10^,^11^.

The subsequent climatic and biotic developments during the Oligocene and early Miocene in Central Asia have received less attraction, although these epochs saw dramatic shifts in global climate, expressed by major ice sheet oscillations in the southern Hemisphere^12^,^13^,^14^,^15^,^16^. Starting with the O1 glacial, the Oligocene was characterized by recurring glacial episodes, roughly coinciding with the ~1.2-My-long obliquity cycle^14^,^17^. This astronomically forced pattern was interrupted by a severe cold phase during the mid-Oligocene, framed by the glacial Oi2a and Oi2b isotope events. Geodynamic events may thus have played an additional important role for the development of the Oligocene Glacial Maximum (OGM). This cool phase was followed by the global Late Oligocene Warming (LOW), which was interrupted only by the minor isotope event Oi2c. Though widely recognized in various marine and continental settings^13^,^18^,^19^,^20^, the cause of this warming is still under debate and in Antarctica no warming occurred at all during the late Oligocene^21^. The LOW was rapidly ended by the Mi1 glacial^22^, marking the Oligocene-Miocene transition. As for the Oligocene glacials, the timing of the Mi1 glacial suggests strong forcing by orbital cycles^23^,^24^. Antarctic ice sheets probably expanded up to its present-day configuration during this severe glacial, which lasted ~400 kyr^24^. In Central Asia the onset of the Miocene is thought to have coincided with a renewed pulse of aridification and widespread desert formation, based on sedimentological evidence^25^,^26^,^27^. So far, the influx of these climatic changes on the Central Asian biota was discussed only very briefly and with focus on the EOT^7^; a statistical approach and quantified data were lacking completely. Herein, we present the first comprehensive analysis of mammal diversity (as total number of species per time slice) and turnover rates (differences of species compositions among consecutive samples) during Oligocene and early Miocene times in Mongolia. Based on occurrence and abundance data of 175 mammal species (128 small mammals, 47 large mammals), we try to detect fits and misfits with global and regional climate developments. In addition, paleosol carbonate samples were taken and high-resolution magnetic susceptibility measurements were carried out during logging, to examine additional climate proxies.

## Geological setting

**Figure 1:**
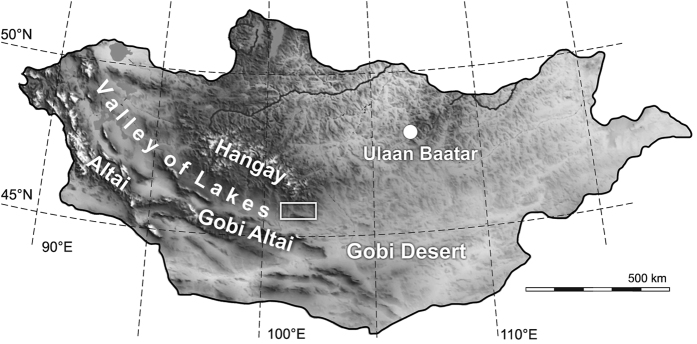
Geographic map of Mongolia, showing the position of the investigation area (white frame), between the Hangay range and the Gobi Altai Mountains. Created with the software CorelDRAW, version X7, http://www.coreldraw.com/de/product/home-student/); modified from https://commons.wikimedia.org/wiki/File:Mongolia_Topography.png; base map source: https://upload.wikimedia.org/wikipedia/commons/f/fe/Mongolia_Topography.png.

**Figure 2:**
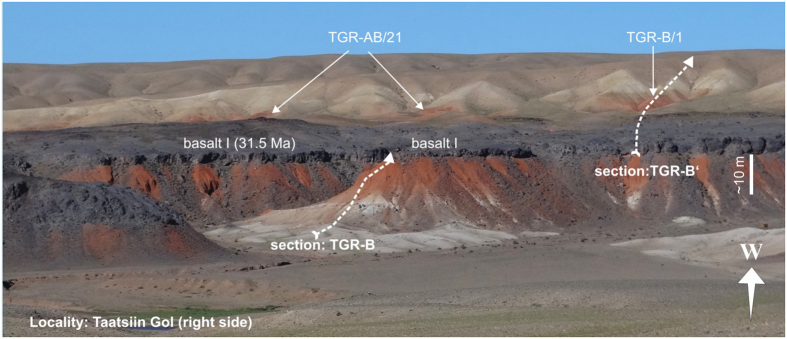
Typical outcrop situation in the Taatsiin Gol area, showing the whitish fluvial-lacustrine deposits of the Eocene Tsagaan Ovoo Formation overlain by the brick-red lower Oligocene Hsanda Gol Formation with the widespread basalt I.

The Mongolian Valley of Lakes is an elongate intramontane depression situated between the Gobi Altai Mountains in the west and southwest and the Hangay range in the northeast (Fig. 1). Terrestrial sedimentation commenced during the Cretaceous and continued up to the Quaternary with major interruptions during the Paleocene, early Eocene and Pliocene^28^. The Oligocene and lower Miocene deposits are united in the Hsanda Gol Formation (HGF) and the Loh Formation (LF). Both are widely exposed in the Taatsiin Gol Basin and extraordinarily rich in fossil mammals (Fig. 2). The Oligocene Hsanda Gol Formation is lithologically rather monotonous, being dominated by reddish clays and silts with numerous caliche and paleosol layers; sand and gravel lenses are rare. A huge basalt flow separates the formation and reduced the early Oligocene paleorelief^29^. The depositional environment was interpreted as semiarid, open steppe with ephemeral rivers and playa lakes with primarily dust deposition based on sedimentological data^7^,^29^. The upper Oligocene to Miocene Loh Formation is partly interfingering with the HGF and lithologically more diversified. It exposes brownish-reddish to greyish clay and silt with caliche layers. Intercalations of fluvial cross-bedded sand and gravel are frequent. Faulting processes and increased uplift of the mountain ranges north and south of the basin increased paleorelief during the late Oligocene and Miocene^29^.

A detailed description of the regional geology of the area is given in Höck *et al*.^28^; lithological logs of the sections are provided in the Supplementary Fig. 1. An absolute dating of our sections based on intercalated flood basalts^28^ and magnetostratigraphic dating^7^ allow a precise correlation with the marine record.

## Results

**Figure 3:**
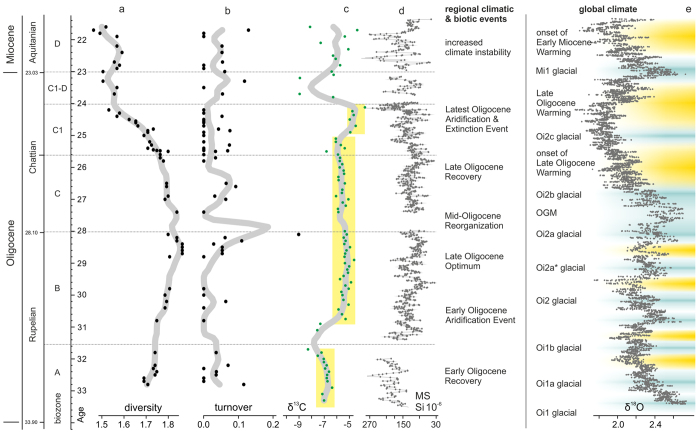
Total diversity of mammal species (**a**) and turnover rates (**b**) during the Oligocene and early Miocene in the Valley of Lakes (Mongolia) compared to composite curves of δ^13^C values of paleosol carbonate (**c**) and magnetic properties of the fossil-bearing sections (**d**); thick grey lines represent Nadaraya–Watson kernel regression estimates, calculated using a bandwidth of 0.5. Major events in regional climate and biotic response are indicated along with the global climate evolution based on the deep-sea benthic foraminiferal oxygen-isotope curve of Zachos *et al*.^16^ with glacial events after Pekar *et al*.^12^,^13^ and Wade & Pälike^17^ (**e**) (OGM = Oligocene Glacial Maximum). Yellow bars in **c** indicate stepwise aridification.

**Figure 4:**
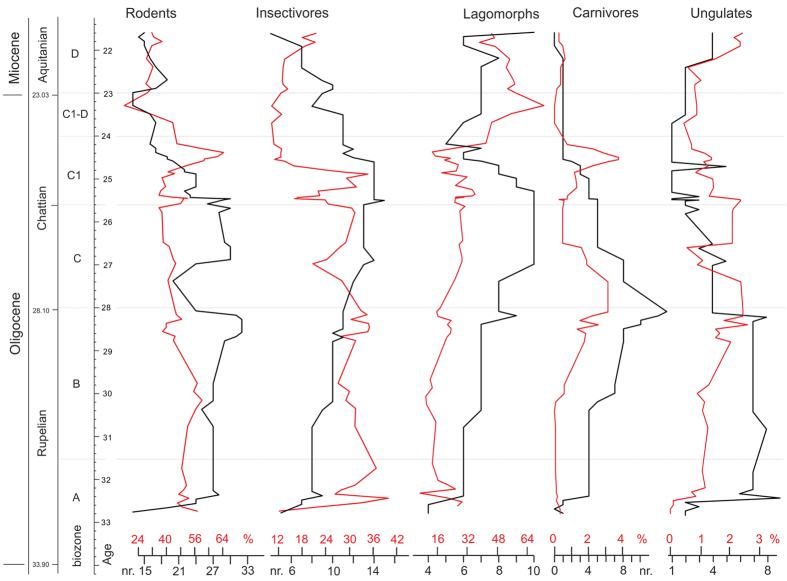
Species-level diversity of major taxonomic groups and their relative abundance (black line = species numbers with range-through assumption; red line = specimens per sample in %, 5-point running mean). Note that each specimen was counted as 1 without any corrections for skeletal element abundance.

A total of 175 species-level taxa are recorded in the Oligocene and lower Miocene samples. Rodentia, Lagomorpha and Eulipotyphla (= “insectivores”) are most speciose and dominate the spectra in species numbers and in counted specimens. Diversity and the temporal distribution of the species reflect several shifts in faunal composition (Figs 3 and 4).

A first diversification trend is observed through biozones A and B (Mann-Kendall trend test: τ = −0.739, p < 0.001) culminating in a maximum diversity of 74 species in the terminal biozone B roughly between 29 to 28 Ma. Despite this slight trend, the overall diversity was rather stable with an average of 57 species (σ = 13, n = 21) throughout the early Oligocene. Rodents dominated the Rupelian assemblages and the end-Rupelian diversity peak is mainly related to the contribution by rodents and to a lesser degree by lagomorphs. The overall increasing trend is also also observed in the insectivore data. Relatively high and stable diversities are also developed by the large herbivores. Carnivores display a constant increase in diversity, which culminates in the late biozone B. Hence, Central Asian mammal faunas experienced an optimum phase between c. 29 to 28 Ma.

After the end-Rupelian peak, a slight drop in diversity occurred during the earliest Chattian between 28 to 27 Ma (early biozone C) coinciding with a major turnover, which is called hereafter the Mid-Oligocene Reorganization (MOR). This drop in diversity is most pronounced in rodents, which lost 32% of their diversity. Simultaneously, large herbivores were strongly reduced and within the carnivores a phase of decline started. Lagomorphs, in contrast, were little affected and insectivores even increased in diversity.

Subsequently, diversities increased again during the early Chattian (biozone C) and ranged around 62 species (σ = 4.5, n = 7). Rodents are prominently represented again and insectivores and lagomorphs attain highest species numbers but the decline of large mammals continued.

From 25.6 to 24 Ma, during the mid-Chattian biozone C1, a major change in diversity patterns occurred. The total diversity dropped significantly from 65 species to 34 representing a diversity loss of 48% (Kendall’s τ = 0.839, p < 0.001). This severe drop was caused by a rapid decline of rodent- and lagomorph-diversities, which was accelerated around 25 Ma. During this event, here termed the Late Oligocene Extinction Event (LOEE), rodents became reduced by 46.6% and lagomorphs by 54.4%. Insectivores were less affected, losing only 26.6% in species diversity but reflect an abrupt reduction in specimen-numbers. Rodents, in contrast, developed a short acme in specimens despite the reduced species number. At the end of this phase large mammals have nearly completely vanished.

Thereafter, diversities remained rather stable at a low level of about 34 species (σ = 3.0, n = 13) throughout the terminal Oligocene and early Miocene (biozones C1-D, D; Kendall’s τ = 0.135, p = 0.575). A minor drop in species richness occurred close to Oligocene/Miocene boundary in rodents and insectivores. The decreasing diversity trend of biozone C1 continued in insectivores, whilst lagomorphs became slightly more speciose again. This lagomorph recovery is even more pronounced in specimen numbers. Within the large mammals only the herbivores recovered gradually during the early Miocene, while carnivores remained very rare.

The geochemical and geophysical data display also several roughly parallel trends. Between 34 and 32 Ma (biozone A) δ^13^C values range around −7.0‰ (σ = 0.5, n = 14). After a short shift towards depleted values around 31.5 − 31.0 Ma, rather stable values with a mean of −5.5‰ (σ = 0.5, n = 45) occur from 30.8 to 25.8 Ma, throughout biozones B and C. A further step towards heavier values around −5.2‰ (σ = 0.6, n = 20) is observed in biozone C1, covering an interval from 25.8 to 24.0 Ma. The trend is reversed between 24 and 23 Ma, in biozone C1-D, with strongly fluctuating and overall more depleted values with a mean of −5.8‰ (σ = 1.6, n = 12). This fluctuating pattern continues into the early Miocene with more heavy values and a mean of −6.0‰ (σ = 0.9, n = 19).

The Oligocene deposits are distinguished from the underlying fluvial Eocene Tsagaan Ovoo Formation in the abrupt increase in magnetic susceptibility, from values around 35 (σ = 20.0, n = 58) (not shown in Fig. 3) to a mean of 180.2 (σ = 43.5, n = 110) in the lower Hsanda Gol Formation. Biozone B displays a slight drop to a mean of 122.5 (σ = 36.9, n = 468) with moderate fluctuations. This pattern continues until about 24.6 Ma with a mean of 120.2 (σ = 33.9, n = 442). From 24.6 Ma onwards, spanning the uppermost biozone C1 and C1-D, the MS signal is strongly fluctuating and has a higher mean value of 147.6 (σ = 52.1, n = 205). The fluctuating MS signal continuous into the early Miocene and the mean MS value shifts to 167.8 (σ = 117.3, n = 157).

## Discussion

During the Oligocene the Taatsiin Gol Basin was part of the broad arid/semiarid climate belt extending in W-E direction through Central Asia^26^. Intensified uplift of the Tibetan plateau and the Hangay Mountains, as well as, and the prevailing westerlies which carry low moisture, amplified aridity^10^,^11^,^30^. Based on sedimentological evidence, the depositional environments in the Taatsiin Gol Basin is interpreted as semiarid open steppe with ephemeral rivers and playa lakes^7^. With the onset of the Miocene, the Neogene monsoon system became established and the Central Asian arid/subarid belt shrunk due to increased humidification in southeast and southwest of China^26^. The Taatsiin Gol Basin, however, remained part of the steppe region. In such semiarid regions, magnetic susceptibility in paleosols and weathered aeolian deposits are enhanced by *in situ* formation of new ferrimagnetic minerals^31^,^32^. This process causes a positive relationship between magnetic susceptibility and annual rainfall in loess and paleosols^32^. The relationship is based on the fact that higher precipitation increases chemical weathering and biological productivity on the surface, which support pedogenesis. Given the assumed semiarid conditions in the Valley of Lakes during the Oligocene, the fluctuations of the magnetic properties observed in the Hsanda Gol and Loh formations may reflect at least partly changes in climate and especially in precipitation. A comparable climate-sensitivity is documented for δ^13^C values in soil carbonates. Carbon isotopes in the Eocene to Upper Miocene section of the Valley of Lakes have been studied by Caves *et al*.^11^, who identified two main mechanisms to explain the observed increase in δ^13^C in paleosol carbonates. These are a decrease in primary productivity due to increasing aridity and an increase in evaporation coupled with lowered precipitation. A significant contribution by C4 plants, which would also trigger higher δ^13^C-values, can be ruled out for the studied sections. C4 plants did not expand to Mongolia before late Miocene times^33^ and the Oligocene paleosols of the Valley of Lakes predate the rise of this photosynthesis-pathway clearly by about 15–20 Million years^34^,^35^.

This climate-sensitivity is also reflected by the MS and δ^13^C data from the Taatsiin Gol Basin. Both proxies document stable conditions immediately after the Oi1 glacial during the earliest Oligocene recovery phase. This phase shows relatively depleted δ^13^C values and relatively high MS values pointing to moderately arid conditions. At c. 31.0 Ma a distinct positive shift in δ^13^C occurs marking the Early Oligocene Aridification Event (EOAE) in Central Asian climate. This event coincides with the Oi1b glacial and marks the boundary between biozones A and B^28^,^29^. The mammal assemblages do not show a significant change in diversities and the family spectra remain similar across the EOAE; similarly, turnover rates remained low. Overall, the mammal communities were able to buffer the climate change. Moreover, after the EOAE, mammal communities start to diversify and attain an optimum during the late Rupelian. Along with a large number of ground-dwelling small mammals, the communities are characterized also by numerous ungulates and several carnivores. A Serengeti-like landscape rather than an arid desert developed in the Valley of Lakes. This optimum was most probably related to reduced aridity stress, which allowed the development of ample vegetation supporting herds of large mammals.

After this optimum, a slight drop of diversities occurred around 28 Ma, mainly caused by a loss of rodents. Although the change in total diversity is rather moderate, the turnover rate is very high. Large herbivores were strongly reduced and carnivores started to decline. This Mid-Oligocene Reorganization (MOR) is not reflected in the available geochemical and geophysical records, which suggest stable conditions in a semiarid depositional environment. The coincidence with the timing of the Oligocene Glacial Maximum, however, is conspicuous. Hence, a change in climate parameter, such as temperature and/or seasonality, not detected in our proxy data, might have triggered the turnover.

During the middle Chattian rather stable conditions became established again; small mammals developed high species diversities whereas large mammals remained rare. This moderate recovery phase coincided with the onset of the Late Oligocene Warming and ended abruptly around 25.6 Ma. The severe loss in mammal diversity of 48% between 25.6−24 Ma, coupled with fluctuating turnover rates and a general reorganization of mammal communities, is termed herein the Late Oligocene Extinction Event (LOEE). It coincided with a distinct positive shift in δ^13^C values of soil carbonates and the peak of the Late Oligocene Warming. This shift suggests that increased aridity stress might have caused the LOEE, affecting the mammal communities much more than the first aridification step in the early Oligocene. Vegetation was obviously too reduced to maintain larger groups of ungulates; consequently, carnivores disappeared as well.

During the terminal Oligocene, climate became increasingly unstable as indicated by strongly fluctuating MS and δ^13^C values. The depleted δ^13^C values point to periods of increased precipitation, which is also supported by the presence of cross-bedded fluvial deposits in the sections. Terrestrial gastropods from this interval point to more humid settings with a dense vegetation cover of a floodplain or ephemeral lakes^36^. This phase of climate instability resulted in a rodent species and abundance decline and a striking reduction in insectivores specimens in the samples. Only lagomorphs were able to cope with the conditions, indicated by high individual numbers in the samples. This pattern indicates that the transition from the Oligocene Warming towards the Mi1 glacial caused a major climate change in this region of Central Asia. During the Mi1 glacial and the early Miocene the climate instability persisted although the more humid phase was ended.

## Conclusions

The “big chill” at the Eocene/Oligocene Transition (EOT) caused a major transformation of the mammal communities of Central Asia. In the Valley of Lakes, fluvial-lacustrine environments became replaced by a semiarid steppe. Nevertheless, mammal communities recovered quickly during the early Oligocene. An aridification at c. 31.0 Ma step, distinctly postdating the EOT, caused some turnover, but no loss in diversity which was constantly rising throughout the early Oligocene. During this optimum phase, Serengeti-like mammal communities with large herbivores and several carnivores developed. The positive trend was abruptly ended at c. 28 Ma when turnover rates peaked and especially rodents declined significantly. This Mid-Oligocene Reorganization in Central Asia coincided with the global Oligocene Glacial Maximum. Aridity remained constant during the OGM, as suggested by paleosol carbonate isotopes. Therefore, the global cooling might have been the driving force for the turnover. A climate related change of vegetation would also explain the loss in large herbivores. A recovery of the Central Asian small mammal communities was initiated by the onset of the Late Oligocene Warming (LOW). However, this bounce back, was curbed drastically during the peak of the LOW. Paleosol carbon values indicate a second phase of aridification around 25 Ma and a dramatic decline of large mammal communities. A synchronous faunal turnover took place across Asia (e.g. Ulantatal in northern China^37^,^38^ and even Europe^39^ suggesting a continental scale event of lowered precipitation. During the waning LOW, precipitation increased again during the terminal Oligocene but the mammal communities could not recover and larger mammals remained scarce. A third aridification event occurred at the Oligocene/Miocene boundary coinciding with the Mi1 glacial. Strongly fluctuating geochemical and geophysical signals suggest unstable climate conditions, which are also reflected in high turnover rates in mammal communities.

These patterns document a strong biotic feedback in this part of Central Asia to global climate change. A first crisis was caused by the cooling during the Oligocene Glacial Maximum but the even more severe breakdown of diversities was caused by the amplified aridification during the Late Oligocene Warming. Due to the already considerable loss during the Late Oligocene Extinction Event, the subsequent Mi1 glacial had surprisingly little impact on overall mammal diversity. Our data document that the post-Eocene aridification of Central Asia happened in several steps, was interrupted by short episodes of increased precipitation, and was not a gradual process. The observed patterns cast doubts on a gradual and purely tectonically induced climate change in Central Asia and underlines the impact of global climate dynamics on regional faunas.

Interestingly, the big diversity loss in Central Asian mammal faunas coincided with a global warming phase, whereas global cooling rather initiated faunal turnover. The question, why the cooling at the EOT and the warming in the late Oligocene both boosted aridification in Central Asia remains open to climate modelers.

Due to the spotty data from Chinese regions^38^ a direct comparison of the herein described trends is difficult. Thus, future work will be needed to provide comparable data from other Central Asian regions to distinguish between regional trends, restricted to the Valley of Lakes, and continental scale developments.

## Methods

In total, 22 natural outcrops were studied in the Taatsiin Gol Basin (see Supplementary Table 1 for localities, coordinates and section acronyms as used for samples). The sections cover the Hsanda Gol Formation and the Loh Formation including its lower Miocene parts. All sections were logged in detail concerning lithology and sedimentological structures. Within the sections more than 90 fossil bearing horizons were detected. The specimens analyzed herein derive from 40 stratified samples of the HGF and from 20 samples from the LF. The stratigraphic position of the samples is inferred from their relative positions within the sections and stratigraphic tie points provided by radiometric datings^28^, magnetostratigraphy^7^ and biostratigraphy^28^,^29^,^40^. For better visualization, the sample positions are plotted in time domain in Fig. 3 (see Supplementary Fig. 1 for the exact positions of the samples).

During eight field-campaigns from 1995–2012, bulk samples of one to several tons were taken from these horizons and screened for fossil mammal remains. Subsequently, the samples were split into systematic groups and were identified and quantified by specialists and partly already published in taxonomic papers^41^,^42^,^43^,^44^,^45^. The collecting campaigns resulted in a dataset of 18608 identified specimens, representing 175 species-level mammal taxa (Supplementary Table 1). This method clearly focused on small mammals, whereas larger mammals might be underrepresented.

The sampling method allows not only a qualitative taxonomic analysis of the samples but is also suited for quantitative analyses of the assemblages. For the diversity estimates data were transformed into a presence/absence matrix and a diversity curve was computed in PAST 2.17c^46^, based on a range-through assumption, i.e., that all taxa are supposed to have been continuously existing from their first to last appearance^47^ (Fig. 3a). In order to test for significance in qualitatively observed trends, we performed a Mann-Kendall trend test. Due to the fluctuating nature of the diversity time series, we divided it into discrete units that match the diversity increase during the Rupelian (biozones A to B), the slight decrease in the early Chattian (biozone C), the subsequent mid-Chattian strong biodiversity decline (biozone C1) and the following low-diverse interval at the Oligocene–Miocene transition (biozones C1-D to D). Differences of species compositions among samples were evaluated using the Beta-Jaccard dissimilarity measure (β-Jaccard) for pairwise comparisons^48^. To differentiate between species loss and replacement, we calculated the index components for turnover, which is unbiased from richness differences^48^ (Fig. 3b). These analyses were computed in R 3.1.3 using packages betapart v. 1.3^49^ and Kendall v. 2.2^50^.

Along with sediment samples, 117 paleosol carbonate samples were taken, covering the entire Oligocene and the lowermost Miocene (Fig. 3c, Supplementary Table 2). Isotopic analyses were performed in the Stable Isotope Laboratory at the Institute of Earth Sciences, University of Graz, using an automatic Kiel II preparation line and a Finnigan MAT Delta Plus mass spectrometer. Samples were dried and reacted with 100% phosphoric acid at 70 °C. International standard NBS-19 and an internal laboratory standard were analyzed continuously for accuracy control. Standard deviation was less than 0.05‰ for δ^13^C. Isotopic data are reported in conventional δ notation relative to the Vienna Peedee belemnite (V-PDB) standard in ‰ units.

During logging of the sections high-resolution magnetic susceptibility measurements were carried out on site, using a handheld “SM-20” magnetic susceptibility meter (GF Instruments; sensitivity: 10^−6^ SI units). Results are reported in dimensionless SI units (MS) (Fig. 3d, Supplementary Table 3). Layers close to basaltic layers were clearly overprinted by the high MS values of the basalts and therefore were excluded from analysis. Similarly, all topmost layers of the sections showed abrupt increases in MS values close to the surface, which is a result from recent pedogenesis and/or bacterial activity^51^. Therefore, these values have been excluded as well from analysis.

## Additional Information

**How to cite this article**: Harzhauser, M. *et al*. Stepwise onset of the Icehouse world and its impact on Oligo-Miocene Central Asian mammals. *Sci. Rep.* **6**, 36169; doi: 10.1038/srep36169 (2016).

**Publisher’s note:** Springer Nature remains neutral with regard to jurisdictional claims in published maps and institutional affiliations.

## Supplementary Material

Supplementary Information
