# Supplementary material for: Stepwise onset of the Icehouse world and its impact on Oligo-Miocene Central Asian mammals
Source: Sci Rep. 2016 Nov 29;6:36169. doi: 10.1038/srep36169 (PMC5126638; doi:10.1038/srep36169)
Supplement: Supplementary Information [file srep36169-s1.pdf]

Stepwise onset of the Icehouse world and its impact on Oligo-Miocene Central Asian mammals

Mathias Harzhauser*[1], Gudrun Daxner-Höck[2], Paloma López-Guerrero[2,3], Olivier Maridet[4,5], Adriana Oliver[2,6], Werner E. Piller[7], Sylvain Richoz[7], Margarita A. Erbajeva[8], Thomas A. Neubauer[2], Ursula B. Göhlich[2]

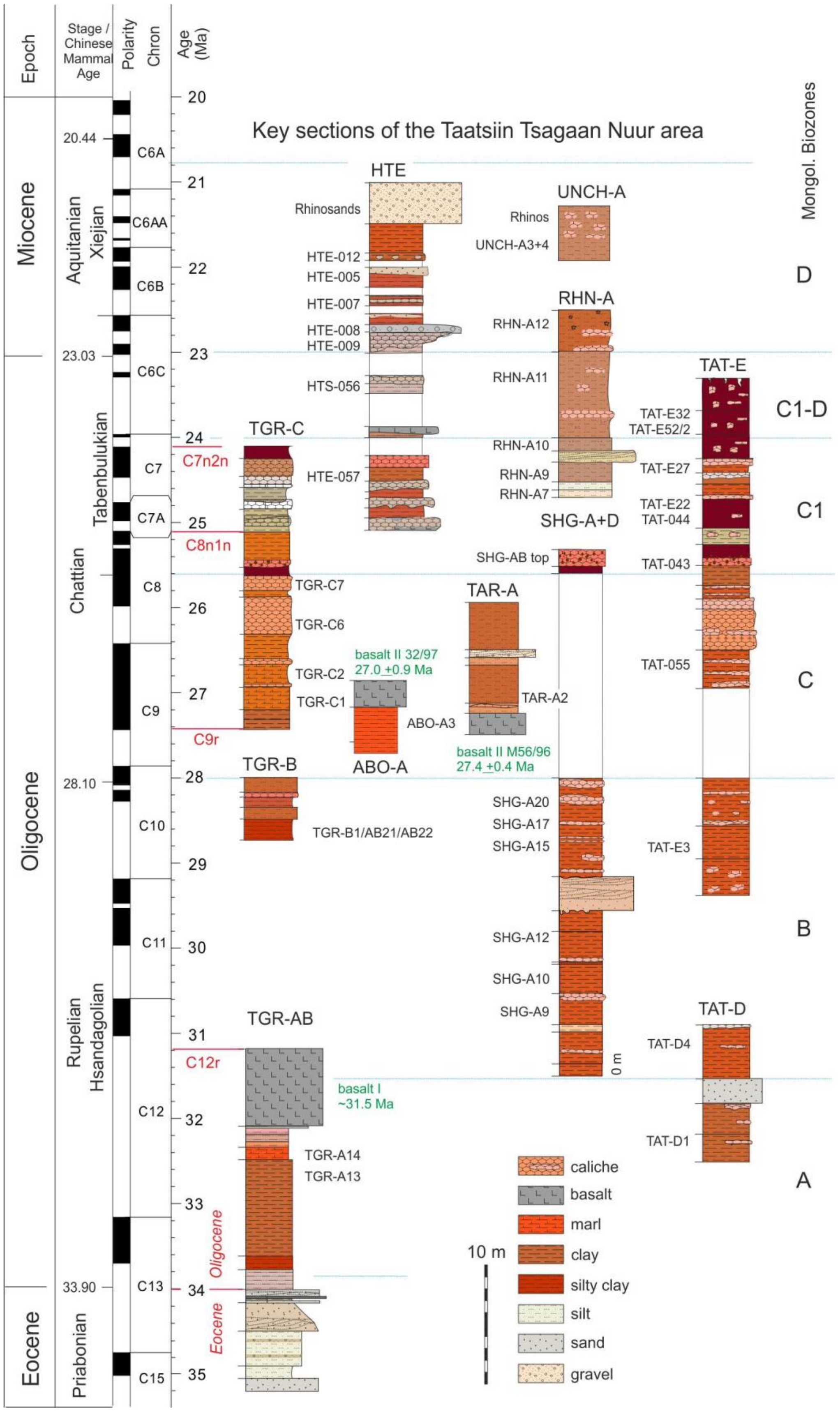

Supplementary Figure 1: Simplified lithologs of the most important studied sections with sample positions.

Supplementary Table 1: Quantitative data on the occurrence and abundance of species-level mammal taxa in the investigated samples. Sample number codes indicate section acronyms used in the supplementary figure 1.

Higher taxonomic categories are indicated by a prefix: La = Lagomorpha, M = Marsupialia, I = Insectivores (Eulipotyphla), R = Rodentia, Cr = Creodonta, Ca = Carnivora, Di = Didymoconidae, A = Artiodactyla, P = Perissodactyla.

AGE	21,6	21,7	21,8	21,9	22,2	22,4	22,7	22,8	22,9	23	23,3	23,5	23,7	24,2	24,3	24,4	24,5	24,55	24,6	24,7
BIOZONE	biozone D	biozone D	biozone D	biozone D	biozone D	biozone D	biozone D	biozone D	biozone D	biozone D	biozone C1-D	biozone C1-D	biozone C1-D	biozone C1	biozone C1	biozone C1	biozone C1	biozone C1	biozone C1	biozone C1
LOCALITY	Unkheltseg	Luuny Yas	Luugar Khudag	Huch Teeg	Hotuliin Teeg	Hotuliin Teeg	Hotuliin Teeg	Hotuliin Teeg	Hotuliin Teeg	Hotuliin Teeg	Huch Teeg	Hotuliin Teeg	Tatal Gol	Huch Teeg	Huch Teeg	Hotuliin Teeg	Hotuliin Teeg	Tatal Gol	Tatal Gol	Huch Teeg
SAMPLE	UNCH-A3+4	LUS-027-029	LOG-A1	RHN-A12,020	HTE-012	HTE*	HTE-005+007	HTE-014-018	HTE-008	HTE-009	RHN-021+22	HTS-056	TAT-E32	RHN-019	RHN-A9+10	HTE-057	HTSE-009+013	TAT-E27	TAT-E22	RHN-023
COORDINATES	N 45°27’40.1” E 101°12’04.4”	N 45° 32’05.5” E 100°56’49.9”	N 45°32’09.6” E 101°00’51.5”	N 45°29’29.9” E 101°12’17.1”	N 45°29’11.9”. E 101°11’49.3”	N 45°29’09.7” E 101°11’48.2”	N 45°29’09.7” E 101°11’49.0”	N 45°29’07.4” E 101°11’58.9”	N 45°29’07.4” E 101°11’58.9”	N 45°29’07.4” E 101°11’60”	N 45°29’30.6” E 101°12’19.1”	N 45°28’53.2” E 101°11’34.9”	N 45°18’12.6’’ E 101°37’15.7”	N 45°29’30.5” E 101°12’20.2”	N 45°29’30.5” E 101°12’20.4”	N 45°28’54.2” E 101°12’26.2”	N 45°29’07.4” E 101°11’58.9”	below TAT-E32	below TAT-E33	N 45°29’33.6” E 101°12’30.0”
La_Ordolagus teilhardi	0	0	0	0	0	0	0	0	0	0	0	0	0	0	0	0	0	0	0	0
La_Desmatolagus vetustus	0	0	0	0	0	0	0	0	0	0	0	0	0	0	0	0	0	0	0	0
La_Desmatolagus youngi	0	0	0	0	0	0	0	0	0	0	0	0	0	0	0	0	0	0	0	0
La_Desmatolagus gobiensis	0	0	0	0	0	26	0	0	0	0	0	0	0	0	0	0	0	0	0	0
La_Desmatolagus robustus	0	0	0	0	0	0	0	0	0	0	0	0	0	0	1	0	0	0	0	0
La_Desmatolagus simplex	0	0	0	0	0	0	0	0	0	0	0	0	0	0	0	0	0	0	0	0
La_Desmatolagus shargaltensis	0	0	0	0	0	0	0	0	0	0	0	0	0	0	0	0	0	0	0	0
La_Desmatolagus chinensis	0	0	0	0	0	0	0	0	0	0	0	0	0	0	0	0	0	0	0	0
La_Desmatolagus orlovi	0	0	0	0	0	0	0	0	0	0	0	0	0	0	0	0	0	0	0	0
La_Desmatolagus sp.	12	0	0	0	8	0	5	0	0	0	0	0	0	0	0	0	0	0	0	0
La_Bohlinotona pusilla	0	0	0	0	0	0	0	0	0	0	0	0	0	0	6	0	0	0	0	0
La_Sinolagomys kansuensis	0	0	0	6	0	7	4	99	104	0	38	67	20	24	43	0	0	16	0	7
La_Sinolagomys major	2	0	0	0	0	78	0	0	47	0	0	0	1	9	0	0	4	0	8	0
La_Sinolagomys ulungurensis	436	118	17	25	0	171	99	7	23	0	0	4	7	0	0	0	0	0	0	0
La_Sinolagomys sp.	250	110	0	0	0	0	0	0	0	0	0	89	0	55	30	3	0	0	0	0
La_Amphilagus magnus	36	22	0	1	0	12	74	80	22	0	0	5	0	0	0	0	0	0	0	0
La_Amphilagus orientalis	10	42	0	0	0	35	0	0	0	0	0	0	0	0	0	0	0	0	0	0
La_Amphilagus plicadentis	2	0	0	0	0	0	0	0	0	0	0	0	0	0	0	0	0	0	0	0
La_Bellatona kazakhstanica	3	0	0	0	0	0	0	0	0	0	0	0	0	0	0	0	0	0	0	0
La_Bellatona yanghuensis	6	0	0	0	0	0	0	0	0	0	0	0	0	0	0	0	0	0	0	0
La_Alloptox minor	1	0	0	0	0	0	0	0	0	0	0	0	0	0	0	0	0	0	0	0
M_Asiadelphis tjutkovae	0	0	0	0	0	0	0	0	0	0	0	0	0	0	0	0	0	0	0	0
M_Asiadelphis zaissanensis	0	0	0	0	0	0	0	0	0	0	0	0	0	0	0	0	0	0	0	0
I_E_Zaraalestes minutus	0	0	0	0	0	0	0	0	0	0	0	0	0	0	0	0	0	0	0	0
I_Zaraalestes sp.	0	0	0	0	0	0	0	0	0	0	0	0	0	0	0	0	0	0	0	0
I_Exallerix pustulatus	0	0	0	0	0	0	0	0	0	0	0	0	0	0	0	0	0	0	0	0
I_Exallerix sp.	1	0	0	0	0	1	0	0	4	0	0	0	0	0	0	0	0	0	0	0
I_Palaeoscaptor acridens	12	0	0	0	3	0	0	3	10	0	0	0	0	0	0	2	0	0	0	0
I_Palaeoscaptor rectus	0	0	0	0	0	0	0	1	3	2	0	8	0	0	0	0	0	0	3	4
I_Palaeoscaptor gigas	0	0	0	0	0	0	0	0	0	0	0	0	0	0	0	0	0	1	0	0
I_Palaeoscaptor tenuis	0	0	0	1	0	0	0	0	1	0	0	4	0	0	0	0	1	0	0	0
I_Amphechinus taatsiingolensis	0	0	0	11	0	0	0	0	2	0	0	28	2	2	0	0	4	0	0	0
I_Amphechinus minutissimus	0	0	0	0	0	0	0	0	0	1	0	3	0	1	2	1	0	3	5	0
I_Amphechinus major	0	0	0	0	0	0	0	0	0	0	0	16	6	1	1	2	3	5	1	1
I_Amphechinus aff. taatsiingolensis	170	11	12	23	13	0	72	23	11	0	0	0	0	0	0	0	0	0	0	0
I_Erinaceidae indet.	0	5	0	4	0	0	0	0	1	0	0	2	1	0	0	1	0	0	0	1

AGE	24,8	24,85	24,9	25	25,2	25,3	25,4	25,45	25,47	25,48	25,5	25,6	25,7	25,8	26,5	26,6	26,9	27	27,4	28,1
BIOZONE	biozone C1	biozone C1	biozone C1	biozone C1	biozone C1	biozone C1	biozone C1	biozone C1	biozone C1	biozone C1	biozone C1	biozone C1	biozone C	biozone C	biozone C	biozone C	biozone C	biozone C	biozone C	biozone B
LOCALITY	Huch Teeg	Tatal Gol	Loh	Loh	Tatal Gol	Argalatyn Nuruu	Argalatyn Nuruu	Toglorhoi	Tatal Gol	Toglorhoi	Del	Hsanda Gol	Taatsiin Gol	Tatal Gol	Toglorhoi	Toglorhoi	Unzing Churum	Abzag Ovo	Taatsiin Gol	Unkheltseg
SAMPLE	RHN-A7+8	TAT-044	LOH-C1	LOH-B3	TAT-043	IKH-B5	IKH-A5	TGW-A5	TAT-051/2	TGW-A3+4	DEL-B12	SHG-AB>20 top	TGR-C7	TAT-055	TGW-A2a+2b	TGW-A1	TAR-A2	ABO-A3	TGR-C1+2	UNCH-A3B
COORDINATES	N 45°29’36.0” E 101°12’22.2”	N 45°18’00.5’’ E 101°37’20.6”	N 45°17’ 04.9” E 101°47’22.9”	N 45°17’04.9” E 101°47’22.7”	N 45°17’59.8” E 101°37’17.1”	close to IKH-A5	N 45°17’49.1” E 102°05’00.7”	N 45°22’37.6” E 101°05’49.2”	N 45°18’09.4” E 101°37’14.5”	N 45° 22’ 37.6” E 101° 05’ 50.1’’	N 45°27’10.2” E 101°22’21.9”	close to SHG-AB-9	N 45°23’10.9” E 101°14’34.9”	N 45°17’59.0” E 101°37’16.6”	N 45° 22’ 37.6” E 101° 05’ 49.2	N 45° 22’ 37.6” E 101° 05’ 48.0	N 45°31’14.4” E 101°18’19.2”	N 45°34’25.4” E 101°03’49.7”	N 45°24’13.5” E 101°15’53.0	N 45°27’40.1” E 101°12’04.8”
La_Ordolagus teilhardi	0	0	0	0	0	0	0	0	0	0	1	0	0	0	1	0	0	0	0	0
La_Desmatolagus vetustus	0	0	0	0	0	0	0	0	0	0	0	0	0	0	0	0	0	0	0	0
La_Desmatolagus youngi	0	0	0	0	0	0	0	0	0	0	0	0	0	0	0	0	0	0	0	0
La_Desmatolagus gobiensis	4	1	0	0	0	0	0	0	0	23	0	0	0	1	136	0	14	3	472	10
La_Desmatolagus robustus	0	0	0	0	0	0	0	0	0	0	0	0	0	0	0	0	0	0	0	0
La_Desmatolagus simplex	0	0	0	0	0	0	0	0	16	0	20	0	0	111	48	0	0	8	24	60
La_Desmatolagus shargaltensis	0	0	0	0	0	0	0	0	0	0	0	0	0	0	0	0	34	0	0	0
La_Desmatolagus chinensis	7	0	2	0	0	6	0	0	0	0	26	0	13	0	71	0	0	0	24	0
La_Desmatolagus orlovi	0	0	0	0	0	0	0	5	0	18	0	0	0	0	10	0	11	0	84	0
La_Desmatolagus sp.	0	0	9	0	4	0	0	3	0	0	0	0	0	0	85	5	0	0	94	24
La_Bohlinotona pusilla	0	0	0	1	0	0	0	0	0	0	25	0	0	0	24	1	8	0	31	0
La_Sinolagomys kansuensis	0	3	15	0	1	0	0	411	14	0	8	0	0	0	0	0	0	0	0	2
La_Sinolagomys major	0	0	0	1	0	0	0	1	1	0	0	0	0	0	0	0	0	0	0	0
La_Sinolagomys ulungurensis	0	0	0	0	0	0	0	0	0	0	0	0	0	0	0	0	0	0	0	0
La_Sinolagomys sp.	7	0	0	0	0	1	1	5	0	0	8	0	0	0	0	0	0	0	1	0
La_Amphilagus magnus	0	0	0	0	0	0	0	0	0	0	0	0	0	0	0	0	0	0	0	0
La_Amphilagus orientalis	0	0	0	0	0	0	0	0	0	0	0	0	0	0	0	0	0	0	0	0
La_Amphilagus plicadentis	0	0	0	0	0	0	0	0	0	0	0	0	0	0	0	0	0	0	0	0
La_Bellatona kazakhstanica	0	0	0	0	0	0	0	0	0	0	0	0	0	0	0	0	0	0	0	0
La_Bellatona yanghuensis	0	0	0	0	0	0	0	0	0	0	0	0	0	0	0	0	0	0	0	0
La_Alloptox minor	0	0	0	0	0	0	0	0	0	0	0	0	0	0	0	0	0	0	0	0
M_Asiadelphis tjutkovae	0	0	0	0	0	0	0	0	0	0	0	0	0	0	0	0	0	0	0	0
M_Asiadelphis zaissanensis	0	0	0	0	0	0	0	0	0	0	0	0	0	0	0	0	0	0	0	0
I_E_Zaraalestes minutus	0	0	0	0	0	0	0	7	2	0	0	0	0	5	0	0	0	0	0	182
I_Zaraalestes sp.	0	0	0	0	0	0	0	0	0	0	0	0	0	0	0	0	0	0	0	0
I_Exallerix pustulatus	0	0	0	0	0	0	0	0	0	0	0	0	0	0	0	0	0	0	2	0
I_Exallerix sp.	0	0	0	0	0	0	0	0	0	0	0	0	0	0	0	0	0	0	0	0
I_Palaeoscaptor acridens	0	0	0	0	5	0	0	0	0	0	1	0	0	10	21	0	2	0	39	20
I_Palaeoscaptor rectus	1	0	5	0	11	0	1	0	3	0	3	0	0	10	0	0	0	0	0	0
I_Palaeoscaptor gigas	0	0	0	0	0	0	0	1	1	1	0	0	0	0	1	0	1	0	5	0
I_Palaeoscaptor tenuis	0	0	0	0	0	0	0	0	13	0	0	0	0	37	1	0	0	0	1	4
I_Amphechinus taatsiingolensis	0	0	0	0	4	0	0	0	1	0	0	0	1	7	582	16	68	1	270	0
I_Amphechinus minutissimus	9	0	9	0	8	15	0	6	6	0	37	0	0	0	0	0	0	0	0	0
I_Amphechinus major	4	0	31	1	6	7	2	4	23	0	144	0	0	0	0	0	0	0	0	0
I_Amphechinus aff. taatsiingolensis	0	0	0	0	0	0	0	0	0	0	0	0	0	0	0	0	0	0	0	0
I_Erinaceidae indet.	0	0	0	0	18	0	0	0	10	0	0	0	0	6	2	0	0	2	0	0

AGE	28,2	28,3	28,4	28,5	28,6	28,7	28,8	29,8	30	30,2	30,4	30,8	31,8	32,2	32,3	32,4	32,5	32,6	32,7	32,8
BIOZONE	biozone B	biozone B	biozone B	biozone B	biozone B	biozone B	biozone B	biozone B	biozone B	biozone B	biozone B	biozone B	biozone A	biozone A	biozone A	biozone A	biozone A	biozone A	biozone A	biozone A
LOCALITY	Hsanda Gol	Hsanda Gol	Taatsiin Gol	Taatsiin Gol	Taatsiin Gol	Argalatyn Nuruu	Hsanda Gol	Tatal Gol	Taatsiin Gol	Hsanda Gol	Tatal Gol	Del	Tatal Gol	Khongil	Talyn Churum	Hsanda Gol	Tatal Gol	Taatsiin Gol	Taatsiin Gol	Tatal Gol
SAMPLE	SHG-AB17-20	SHG-A15-20	TGR-AB22	TGR-AB21	TGR-B1	IKH-A1-4	SHG-AB12+13	TAT-E3	TGL-A11	SHG-A6-9	TAT-C6+7	DEL-B7+8	TAT-037	HL-A1+2	GRAB-II	SHG-C1-2	TAT-C1-3	TGL-A1+2	TGR-A13+14	TAT-D1
COORDINATES	close to SHG-A6-9	N 45° 16’ 01” E 101° 45’ 45”)	N 45°24’41.1” E 101°15’24.9”	N 45°24’41.1” E 101°15’24.7”	N 45°24’47.3” E 101°15’23.2”	N 45°17’48.4” E 102°04’57.2”	close to SHG-A6-9	N 45°18’12.6’’ E 101°37’15.4”	above TGL-A1+2	N 45°29’30.5” E 101°12’20.2”	N 45°18’19.5” E 101°38’025”	N 45°27’10.2” E 101°22’22.3”	N 45°17’54.1” E 101°37’11.7”	N 45°27’40.4” E 101°09’18.5”	N 45°16’50.4” E 101°57’28.4”	N 45° 15’ 49.9” E 101° 43’ 04.9”	N 45°18’19.5” E 101°38’00.0”	N 45°26’57.4’’ E 101°16’20.9”	N 45°25’12.5” E 101°15’44.3”	N 45°17’52.2” E 101°37’18.5”
La_Ordolagus teilhardi	2	0	0	0	0	3	0	0	0	4	0	1	0	0	0	0	0	0	0	1
La_Desmatolagus vetustus	0	0	0	0	0	0	0	0	0	0	0	0	0	1	2	0	0	1	0	2
La_Desmatolagus youngi	0	0	9	0	0	12	5	0	0	0	0	6	0	0	0	9	1	0	0	19
La_Desmatolagus gobiensis	88	36	79	57	38	64	28	0	27	6	62	5	0	0	0	16	22	59	96	290
La_Desmatolagus robustus	0	0	0	2	0	2	0	0	0	0	0	2	0	0	0	0	0	0	0	0
La_Desmatolagus simplex	0	0	0	0	0	0	0	0	0	0	0	11	7	0	0	0	0	0	0	0
La_Desmatolagus shargaltensis	0	0	0	0	0	0	0	0	0	0	0	0	0	0	0	0	0	0	0	0
La_Desmatolagus chinensis	2	0	0	8	0	13	0	0	0	0	0	0	0	0	0	0	0	0	0	18
La_Desmatolagus orlovi	24	0	0	19	7	0	0	0	0	0	0	11	0	0	0	2	5	0	0	59
La_Desmatolagus sp.	0	76	0	0	0	62	0	0	11	0	0	3	0	0	0	110	0	0	6	150
La_Bohlinotona pusilla	0	0	0	0	0	0	0	0	0	0	0	0	0	0	0	0	0	0	0	0
La_Sinolagomys kansuensis	2	0	0	0	0	0	0	0	0	0	0	0	0	0	0	0	0	0	0	0
La_Sinolagomys major	0	0	0	0	0	1	0	0	0	0	0	0	0	0	0	0	0	0	0	0
La_Sinolagomys ulungurensis	0	0	0	0	0	0	0	0	0	0	0	0	0	0	0	0	0	0	0	0
La_Sinolagomys sp.	0	0	0	0	0	0	0	0	0	0	0	0	0	0	0	0	0	0	0	0
La_Amphilagus magnus	0	0	0	0	0	0	0	0	0	0	0	0	0	0	0	0	0	0	0	0
La_Amphilagus orientalis	0	0	0	0	0	0	0	0	0	0	0	0	0	0	0	0	0	0	0	0
La_Amphilagus plicadentis	0	0	0	0	0	0	0	0	0	0	0	0	0	0	0	0	0	0	0	0
La_Bellatona kazakhstanica	0	0	0	0	0	0	0	0	0	0	0	0	0	0	0	0	0	0	0	0
La_Bellatona yanghuensis	0	0	0	0	0	0	0	0	0	0	0	0	0	0	0	0	0	0	0	0
La_Alloptox minor	0	0	0	0	0	0	0	0	0	0	0	0	0	0	0	0	0	0	0	0
M_Asiadelphis tjutkovae	0	0	0	0	0	0	0	0	0	0	0	0	0	0	0	0	0	0	0	1
M_Asiadelphis zaissanensis	0	1	5	3	2	0	0	0	0	0	0	0	0	0	1	0	0	0	0	0
I_E_Zaraalestes minutus	0	19	313	348	315	25	11	22	76	16	46	27	54	14	7	62	154	91	67	201
I_Zaraalestes sp.	0	0	0	3	0	0	0	0	0	0	0	1	0	0	0	0	0	0	0	0
I_Exallerix pustulatus	0	0	0	0	0	0	0	0	0	0	0	0	0	0	0	0	0	0	0	0
I_Exallerix sp.	0	0	0	0	0	0	0	0	0	0	0	0	0	0	0	0	0	0	0	0
I_Palaeoscaptor acridens	1	8	78	75	34	4	8	5	11	0	16	2	5	1	0	3	9	2	4	13
I_Palaeoscaptor rectus	0	0	0	0	0	0	0	0	0	0	0	0	0	0	0	0	0	0	0	1
I_Palaeoscaptor gigas	0	0	0	0	0	0	0	0	0	0	0	0	0	0	0	0	0	0	0	0
I_Palaeoscaptor tenuis	117	22	4	1	0	0	0	2	1	0	12	0	0	0	0	0	4	1	4	15
I_Amphechinus taatsiingolensis	0	0	0	0	0	0	0	0	0	0	0	0	0	0	0	0	0	0	0	0
I_Amphechinus minutissimus	0	0	0	0	0	0	0	0	0	0	0	0	0	0	0	0	0	0	0	0
I_Amphechinus major	0	0	0	0	0	0	0	0	0	0	0	0	0	0	0	0	0	0	0	0
I_Amphechinus aff. taatsiingolensis	0	0	0	0	0	0	0	0	0	0	0	0	0	0	0	0	0	0	0	0
I_Erinaceidae indet.	0	0	0	0	0	0	0	2	0	0	0	0	9	3	0	0	1	0	0	1

I_Gobisorex kingae	0	0	0	0	0	0	0	0	0	0	0	0	0	0	0	0	0	0	0	1	0	0	0	0	0	1	1	0	0	0	0	0	0	14	0	0	0	0	2	4	4	1	2	0	1	4	0	0	0	0	0	1	1	0	0	1	0	0	2	6
I_Heterosoricinae indet.	0	0	0	0	1	0	0	0	0	0	0	0	0	0	0	0	0	0	0	0	2	0	0	0	0	0	0	0	0	0	0	0	0	0	0	0	0	0	0	0	1	0	0	1	0	0	0	0	0	0	0	0	0	0	0	0	0	0	0	0
I_Taatsiinia hoeckorum	0	0	0	0	0	0	0	0	0	0	0	2	0	0	0	0	0	0	0	0	0	0	0	0	0	0	0	0	0	0	0	0	0	0	0	0	0	0	0	0	3	1	0	0	7	0	0	0	0	0	0	0	0	0	0	0	0	0	0	0
I_Tavoonya altaica	0	0	0	0	0	0	0	0	0	0	0	0	0	0	7	0	0	0	0	0	0	0	0	0	0	0	0	0	0	0	0	0	0	0	0	0	0	0	0	0	0	0	0	0	0	0	0	0	0	0	0	0	0	0	0	0	0	0	0	0
I_Crocidosoricinae indet.	25	0	0	0	1	0	0	0	2	0	0	0	0	0	0	0	0	0	0	0	0	0	0	0	0	0	0	0	0	0	5	0	0	0	2	0	0	0	4	3	0	1	4	2	0	0	0	0	0	0	2	0	0	0	0	0	0	0	0	0
I_Mongolopala tathue	0	0	0	0	0	0	0	0	0	0	0	0	0	0	0	0	0	0	0	0	0	0	0	0	2	0	0	0	0	0	0	0	0	0	0	0	0	0	0	0	0	0	0	0	0	0	0	0	0	0	0	0	0	0	0	4	0	1	0	5
I_Talpidae indet.	0	0	3	0	0	2	1	0	0	0	0	0	0	0	0	0	0	0	0	0	0	0	0	0	0	0	0	6	0	0	0	0	0	0	1	0	0	0	0	0	0	0	0	3	0	0	0	0	0	0	0	0	0	0	0	2	0	0	0	2
I_Asiapternodus mackennai	0	0	0	0	0	0	0	0	0	0	0	0	0	0	0	0	0	0	0	0	0	0	0	0	0	0	0	0	0	0	0	0	0	0	0	0	0	0	0	0	0	0	0	0	0	0	0	0	0	0	0	0	0	0	0	0	0	0	0	1
R_Ninamys arboraptus	0	0	0	0	0	0	0	0	0	0	0	0	0	0	0	0	0	0	0	0	0	0	0	0	0	0	0	0	0	0	0	0	0	0	0	1	0	0	0	1	0	0	1	5	3	5	0	0	1	0	0	0	0	1	0	2	3	0	0	0
R_Ninamys kazimierzi	0	0	0	0	0	0	0	0	0	0	0	0	0	0	0	0	0	0	0	0	0	0	0	0	0	0	0	0	0	0	0	0	0	0	0	0	0	0	0	1	0	0	0	4	1	1	0	0	1	0	1	7	0	0	0	0	0	1	1	1
R_Prosciurus mongoliensis	0	0	0	0	0	0	0	0	0	0	0	0	0	0	0	0	0	0	0	0	0	0	0	0	0	0	0	0	0	0	0	0	0	0	0	0	0	0	0	1	0	0	0	0	0	0	0	0	0	0	0	0	0	0	0	0	0	0	0	2
R_Promeniscomys sinensis	0	0	0	0	0	0	0	0	0	0	0	0	0	0	0	0	0	0	0	0	0	0	0	0	0	0	0	0	0	0	0	0	0	0	0	0	0	0	0	0	0	0	0	0	1	1	0	1	0	0	0	0	0	0	0	0	0	0	0	0
R_Proansomys aff. dureensis	0	0	0	0	0	0	0	0	0	0	0	0	0	0	0	0	0	0	0	0	1	0	0	0	0	0	0	1	0	0	0	0	7	0	11	0	0	0	8	0	0	0	0	0	0	0	0	0	0	0	0	0	0	0	0	0	0	0	0	0
R_Ansomyinae indet.	0	0	0	1	0	0	0	0	0	0	0	0	0	0	0	0	0	0	0	0	0	0	0	0	0	0	0	0	0	0	0	0	0	0	0	0	0	0	2	0	0	0	0	0	0	0	0	0	0	0	0	0	0	0	0	0	0	0	0	0
R_Plesiosciurus aff. sinensis	1	0	0	0	0	0	0	0	1	0	0	0	0	0	0	0	0	0	0	0	0	0	0	0	0	0	0	0	0	0	0	0	0	0	1	0	0	0	0	0	0	0	0	0	0	0	0	0	0	0	0	0	0	0	0	0	0	0	0	0
R_Kherem shandgoliensis	3	0	0	0	0	3	3	0	0	0	0	0	0	0	0	0	0	0	0	0	0	0	1	0	0	0	0	0	0	0	0	0	0	0	0	0	0	0	0	0	0	0	0	0	0	0	0	0	0	0	0	0	0	0	0	0	0	0	0	0
R_Pteromyini indet.	4	0	0	0	0	0	0	0	0	0	0	0	0	0	0	0	0	0	0	0	0	0	0	0	0	0	0	0	0	0	0	0	0	0	0	0	0	0	0	0	0	0	0	0	0	0	0	0	0	0	0	0	0	0	0	0	0	0	0	0
R_Eutamias sp.	2	0	0	0	0	0	0	0	1	0	0	0	0	0	0	0	0	0	0	0	0	0	0	0	0	0	0	0	0	0	0	0	0	0	0	0	0	0	0	0	0	0	0	0	0	0	0	0	0	0	0	0	0	0	0	0	0	0	0	0
R_Eomys orientalis	0	0	0	0	0	0	0	0	0	0	0	0	0	0	0	0	0	0	0	0	0	0	0	0	0	0	0	0	0	0	0	0	0	0	0	0	0	0	0	0	0	0	0	0	0	0	0	0	0	0	0	0	0	0	0	0	1	0	0	0
R_Eomys aff. orientalis	0	0	0	0	0	0	0	0	0	0	0	0	0	0	0	0	0	0	0	0	0	0	0	0	0	0	0	0	0	0	0	0	0	0	0	0	0	0	0	0	3	7	1	5	3	0	0	0	0	0	0	0	0	0	0	0	0	0	0	0
R_Eomys sp.	0	0	0	0	0	0	0	0	0	0	0	0	0	0	0	0	0	0	0	0	0	0	0	0	0	0	0	0	0	0	0	0	0	0	0	0	0	0	0	0	0	0	0	3	0	0	0	0	0	0	0	0	0	0	0	0	0	0	0	0
R_Asianeomys bolligeri	0	0	0	0	0	0	0	0	0	0	0	0	0	0	0	0	0	0	0	0	0	0	0	0	0	0	0	0	0	0	0	0	0	0	11	0	0	0	2	0	0	0	0	0	0	0	0	0	0	0	0	0	0	0	0	0	0	0	0	0
R_Asianeomys dangheensis	10	1	0	0	4	0	28	0	0	0	0	0	2	0	0	0	0	0	0	0	1	0	0	0	0	0	0	0	0	0	2	0	0	0	0	0	0	0	0	0	0	0	0	0	0	0	0	0	0	0	0	0	0	0	0	0	0	0	0	0
R_Karakoromys decessus	0	0	0	0	0	0	0	0	0	0	0	0	0	0	0	0	0	0	0	0	0	0	0	0	0	0	0	0	0	0	0	0	0	0	0	0	0	0	0	0	0	0	0	0	0	0	0	0	0	10	0	0	1	11	0	2	0	4	9	15
R_Huangomys frequens	0	0	0	0	0	0	0	0	0	0	0	0	0	0	0	0	0	0	0	0	0	0	0	0	0	0	0	0	0	0	0	0	0	0	0	0	0	0	0	20	0	8	45	58	44	0	0	0	0	0	0	0	0	0	0	0	1	0	0	0
R_Yindirtemys shevyrevae	0	0	0	0	0	0	0	0	0	0	0	0	0	0	0	0	0	0	0	0	0	0	0	0	0	0	0	0	0	0	0	0	0	0	0	0	0	3	0	0	2	2	2	6	3	0	0	0	0	0	0	0	2	0	0	0	0	0	0	0
R_Tataromys sigmodon	0	0	0	0	0	0	0	0	0	0	0	0	0	0	0	0	1	0	0	0	0	0	0	0	0	0	0	0	0	0	0	0	0	6	42	0	2	0	3	0	0	0	0	3	0	0	0	0	0	0	0	0	0	0	0	0	0	0	0	0
R_Tatataromys minor longidens	0	0	0	0	0	0	0	0	0	0	0	0	0	0	0	0	0	0	0	0	0	0	0	0	0	0	0	0	0	4	21	0	0	2	92	0	14	4	17	0	0	0	0	0	0	0	0	0	0	0	0	0	0	0	0	0	0	0	0	0
R_Tataromys plicidens	0	0	0	0	0	0	0	0	0	0	0	0	0	0	0	0	0	0	0	0	0	0	0	0	0	0	0	0	0	1	1	1	0	2	0	0	0	0	0	0	0	0	0	0	0	0	0	0	0	0	0	0	0	0	0	0	0	0	0	0
R_Yindirtemys aff. ulantatalensis	0	0	0	0	0	0	0	0	0	0	0	0	0	0	0	0	0	0	0	0	0	0	0	0	0	0	0	0	0	0	0	0	0	0	0	0	2	0	0	0	0	0	0	0	0	0	0	0	0	0	0	0	0	0	0	0	0	0	0	0
R_Yindirtemys deflexus	0	0	0	0	0	0	0	0	0	0	0	20	0	0	0	33	65	1	1	59	51	0	21	1	0	13	5	10	18	10	45	0	0	0	0	0	0	0	0	0	0	0	0	0	0	0	0	0	0	0	0	0	0	0	0	0	0	0	0	0
R_Yindirtemys birgeri	0	0	0	0	0	0	0	0	0	0	0	0	0	0	0	0	0	0	0	0	0	0	0	0	0	0	0	0	1	0	0	0	0	0	0	0	0	0	0	0	0	0	0	0	0	0	0	0	0	0	0	0	0	0	0	0	0	0	0	0
R_Yindirtemys suni	4	0	0	0	0	5	0	24	8	9	0	1	1	0	0	0	0	0	0	0	0	0	0	0	0	0	0	0	0	0	0	0	0	0	0	0	0	0	0	0	0	0	0	0	0	0	0	0	0	0	0	0	0	0	0	0	0	0	0	0
R_Prodistylomys sp.	1	0	0	2	5	0	2	0	1	0	0	0	0	0	0	0	0	0	0	0	0	0	0	0	0	0	0	0	0	0	0	0	0	0	0	0	0	0	0	0	0	0	0	0	0	0	0	0	0	0	0	0	0	0	0	0	0	0	0	0
R_Distylomys sp.	4	0	26	2	1	0	0	0	1	0	0	0	0	0	0	0	0	0	0	0	0	0	0	0	0	0	0	0	0	0	0	0	0	0	0	0	0	0	0	0	0	0	0	0	0	0	0	0	0	0	0	0	0	0	0	0	0	0	0	0
R_Ardynomys sp.	0	0	0	0	0	0	0	0	0	0	0	0	0	0	0	0	0	0	0	0	0	0	0	0	0	0	0	0	0	0	0	0	0	0	0	0	0	0	0	0	0	0	0	0	0	0	0	0	0	0	0	0	1	0	0	1	0	2	0	6
R_Anomoemys lohiculus	0	0	0	0	0	0	0	0	0	0	0	0	0	0	0	0	0	0	0	0	0	0	0	0	0	0	0	0	0	0	0	0	0	0	0	0	0	0	0	0	0	0	0	0	0	1	0	0	3	0	0	3	0	0	0	0	0	0	2	0
R_Tsaganomyidae indet.	0	0	0	0	0	0	0	0	0	0	0	2	0	0	0	0	0	0	0	0	0	0	0	0	0	0	0	0	0	0	3	0	0	2	36	5	1	0	68	48	23	23	20	18	60	130	0	0	22	73	14	17	5	3	12	6	10	1	0	65
R_Cyclomylus lohensis	0	0	0	0	0	0	0	0	0	0	0	0	0	0	0	0	0	0	0	0	0	0	0	0	0	0	0	0	0	0	0	0	0	0	0	0	0	0	0	0	8	1	2	0	0	4	0	0	1	4	2	1	0	2	2	0	0	0	0	22
R_Cyclomylus biforatus	0	0	0	0	0	0	0	0	0	0	0	0	0	0	0	0	0	0	0	0	0	0	0	0	0	0	0	0	0	0	0	0	0	0	0	0	0	0	1	0	0	0	0	0	0	0	0	0	0	0	0	0	0	0	0	0	0	0	0	6
R_Cyclomylus intermedius	0	0	0	0	0	0	0	0	0	0	0	0	0	0	0	0	0	0	0	0	0	0	0	0	0	0	0	0	0	0	0	0	1	0	1	0	0	0	0	0	1	6	1	0	0	6	0	0	1	1	0	1	1	0	0	1	4	0	0	9
R_Coelodontomys asiaticus	0	0	0	0	0	0	0	0	0	0	0	0	0	0	0	0	0	0	0	0	0	0	0	0	0	0	0	0	0	0	0	0	0	0	1	0	0	0	5	1	2	1	0	0	1	1	0	0	0	1	0	0	0	0	0	0	1	0	0	15
R_Tsaganomys altaicus	0	0	0	0	0	0	0	0	0	0	0	0	0	0	0	0	4	0	0	0	0	2	0	0	2	2	2	19	4	1	3	0	0	1	2	1	0	0	5	1	3	6	2	2	1	1	0	0	2	0	1	1	0	0	0	0	5	0	0	4
R_Allosminthus khandae	0	0	0	0	0	0	0	0	0	0	0	0	0	0	0	0	0	0	0	0	0	0	0	0	0	0	0	0	0	0	0	0	0	0	0	0	0	0	0	0	0	0	0	0	0	0	0	0	0	0	0	0	0	1	1	2	0	0	5	23
R_Onjosminthus baindi	0	0	0	0	0	0	0	0	0	0	0	0	0	0	0	0	0	0	0	0	0	0	0	0	0	0	0	0	0	0	0	0	0	0	0	0	0	0	0	1	1	1	1	3	4	1	0	0	0	0	0	0	2	0	2	3	0	0	28	0
R_Heosminthus chimidae	0	0	0	0	0	0	0	0	0	0	0	0	1	0	0	0	0	0	3	0	0	0	0	0	5	0	0	0	20	0	0	0	0	20	0	0	6	4	21	150	85	101	240	220	200	78	0	17	25	18	110	120	21	5	7	55	160	100	100	250
R_Heosminthus sp.	0	0	0	0	0	0	0	0	0	0	0	0	0	0	0	0	0	0	0	0	0	0	0	0	0	7	0	0	0	0	0	0	0	5	3	0	0	0	0	4	7	3	0	0	2	4	0	1	0	0	3	1	0	0	0	0	0	0	1	2
R_Shamosminthus sodovis	0	0	0	0	0	0	0	0	0	0	0	0	0	0	0	0	0	0	0	0	0	0	0	0	0	0	0	0	0	0	0	0	0	0	0	0	0	0	0	40	0	0	10	20	20	4	0	3	3	0	0	12	0	0	0	0	3	3	5	9
R_Heosminthus borrae	0	0	0	4	0	0	4	6	0	0	0	0	1	0	0	0	0	3	0	0	0	0	0	0	0	0	0	0	0	0	0	0	2	8	0	0	0	0	0	12	0	0	12	7	3	0	0	0	0	0	1	0	0	0	0	0	0	0	0	0
R_Allosminthus minutus	0	0	0	0	0	0	0	0	0	0	0	0	0	0	0	0	0	0	0	0	0	0	0	0	0	0	0	0	0	0	0	0	0	4	1	1	0	0	0	0	16	5	0	5	3	5	0	0	0	9	4	0	0	0	0	0	0	0	0	0
R_Shamosminthus sp.	0	0	0	0	0	0	0	0	0	0	0	0	0	0	0	0	0	0	0	0	0	0	0	0	0	0	0	0	0	0	0	0	0	0	0	0	0	0	0	0	2	0	0	0	0	0	0	0	0	0	0	0	0	0	0	0	0	0	0	0
R_Shamosminthus tongi	0	0	0	0	0	0	0	0	0	0	0	0	0	0	0	0	0	0	0	0	0	0	0	0	0	0	0	0	0	0	0	0	0	1	0	0	0	0	0	0	0	0	0	0	0	0	0	0	0	0	0	0	0	0	0	0	0	0	0	0
R_Bohlinosminthus parvulus	0	0	0	0	0	0	0	4	3	0	0	4	8	0	0	2	6	14	41	1	15	0	37	0	24	9	0	17	13	0	89	0	8	0	103	2	42	4	81	0	26	0	0	0	0	4	0	0	0	0	0	0	0	2	0	0	8	0	0	0
R_Parasminthus cf. tangingoli	0	0	0	0	0	0	0	0	0	0	0	0	0	0	0	0	0	0	0	0	0	0	0	0	1	0	0	0	0	0	4	0	0	0	5	0	3	0	11	0	0	0	0	0	0	0	0	0	0	0	0	0	0	0	0	0	0	0	0	0
R_Parasminthus debruijni	0	0	0	0	0	0	0	0	0	0	0	0	0	0	1	0	0	1	0	0	0	0	0	0	0	0	0	0	0	0	1	0	0	0	0	0	0	0	4	0	0	0	0	0	0	0	0	0	0	0	0	0	0	0	0	0	0	0	0	0
R_Parasminthus cf.asiaecentralis	0	0	0	0	0	0	0	0	0	0	0	0	0	0	0	0	0	0	0	0	0	0	0	0	0	0	0	0	0	0	2	0	0	0	0	0	1	0	0	0	0	0	0	0	0	0	0	0	0	0	0	0	0	0	0	0	0	0	0	0

R_Plesiosminthus sp.	0	1	0	0	3	0	0	0	0	0	0	0	0	0	0	0	0	0	0	0	0	0	2	0	0	0	0	0	0	0	0	0	0	0	0	0	4	0	0	0	0	0	0	0	0	0	0	0	0	0	0	0	0	0	0	0	0	0	0	0
R_Plesiosminthus asiaticus	0	0	0	0	0	0	0	0	0	0	0	0	0	0	0	0	0	0	0	0	8	0	0	0	0	0	0	0	0	0	0	0	0	0	0	0	0	0	0	0	0	0	0	0	0	0	0	0	0	0	0	0	0	0	0	0	0	0	0	0
R_Plesiosminthus promyarion	0	0	0	0	0	0	0	0	0	0	0	11	0	4	9	0	0	0	0	0	0	0	0	0	0	0	0	0	0	0	0	0	0	0	0	0	0	0	0	0	0	0	0	0	0	0	0	0	0	0	0	0	0	0	0	0	0	0	0	0
R_Heterosminthus cf. lanzhouensis	0	0	0	0	0	0	0	0	0	0	2	7	0	0	0	0	0	0	0	2	0	0	0	0	0	0	0	0	0	0	0	0	0	0	0	0	0	0	0	0	0	0	0	0	0	0	0	0	0	0	0	0	0	0	0	0	0	0	0	0
R_Litodonomys huangheensis	15	0	0	0	0	0	5	0	2	0	0	0	1	0	0	0	0	0	0	0	0	0	1	0	0	0	0	0	0	0	0	0	0	0	0	0	0	0	1	0	0	0	0	0	0	0	0	0	0	0	0	0	0	0	0	0	0	0	0	0
R_Litodonomys lajeensis	17	0	2	6	2	2	25	3	8	0	0	4	4	0	0	0	0	1	1	0	0	0	0	0	1	0	0	0	1	0	0	0	0	0	0	0	0	0	0	0	0	0	0	0	0	0	0	0	0	0	0	0	0	0	0	0	0	0	0	0
R_Heterosminthus firmus	86	0	0	9	0	0	11	3	1	0	0	5	1	1	3	0	0	0	1	0	1	0	3	0	0	0	0	0	0	0	0	0	0	0	0	0	0	0	0	0	0	0	0	0	0	0	0	0	0	0	0	0	0	0	0	0	0	0	0	0
R_Plesiosminthus olzi	0	0	0	0	0	0	4	8	8	0	0	0	0	0	0	0	0	0	0	0	0	0	0	0	0	0	0	0	0	0	0	0	0	0	0	0	0	0	0	0	0	0	0	0	0	0	0	0	0	0	0	0	0	0	0	0	0	0	0	0
R_Plesiosminthus barsboldi	18	0	4	6	4	0	0	0	0	0	0	0	0	0	0	0	0	0	0	0	0	0	0	0	0	0	0	0	0	0	0	0	0	0	0	0	0	0	0	0	0	0	0	0	0	0	0	0	0	0	0	0	0	0	0	0	0	0	0	0
R_Heterosminthus aff. nanus	6	0	0	0	0	0	1	1	0	0	0	0	0	0	0	0	0	0	0	0	0	0	0	0	0	0	0	0	0	0	0	0	0	0	0	0	0	0	0	0	0	0	0	0	0	0	0	0	0	0	0	0	0	0	0	0	0	0	0	0
R_Tachyoryctoides radnai	0	0	0	0	0	0	0	0	0	0	0	0	0	0	0	0	0	0	0	0	0	0	0	0	0	0	0	0	0	1	0	0	0	1	0	0	0	0	12	0	0	0	0	0	0	0	0	0	0	0	0	0	0	0	0	0	0	0	0	0
R_Tachyoryctoides bayarmae	0	0	0	0	0	0	0	0	0	0	0	0	0	0	0	0	0	0	0	0	0	0	0	0	0	0	0	0	0	7	0	0	0	0	0	0	0	0	2	0	0	0	0	0	0	0	0	0	0	0	0	0	0	0	0	0	0	0	0	0
R_Tachyoryctoides obrutschewi	0	0	0	0	0	0	0	0	0	0	0	0	0	0	0	2	0	0	0	0	0	0	0	0	0	1	0	0	1	0	0	1	0	0	0	0	0	0	0	0	0	0	0	0	0	0	0	0	0	0	0	0	0	0	0	0	0	0	0	0
R_Tachyoryctoides tatalgolicus	0	0	0	0	0	0	0	0	0	0	0	0	0	0	0	0	1	0	1	0	0	0	0	0	4	0	0	0	0	0	0	0	0	0	0	0	0	0	0	0	0	0	0	0	0	0	0	0	0	0	0	0	0	0	0	0	0	0	0	0
R_Tachyoryctoides sp.	0	0	0	0	0	0	0	0	0	0	1	0	2	0	0	0	0	1	0	0	0	1	0	0	3	0	0	0	0	0	0	0	0	0	0	0	0	0	0	0	0	0	0	0	0	0	0	0	0	0	0	0	0	0	0	0	0	0	0	0
R_Tachyoryctoides kokonorensis	28	2	0	0	27	70	3	5	0	1	0	0	0	0	0	0	0	0	0	0	0	0	0	0	0	0	0	0	0	0	0	0	0	0	0	0	0	0	0	0	0	0	0	0	0	0	0	0	0	0	0	0	0	0	0	0	0	0	0	0
R_Tachyoryctoides engesseri	0	0	0	0	0	16	0	6	6	1	0	0	0	0	0	0	0	0	0	0	0	0	0	0	0	0	0	0	0	0	0	0	0	0	0	0	0	0	0	0	0	0	0	0	0	0	0	0	0	0	0	0	0	0	0	0	0	0	0	0
R_Ayakozomys sp.	0	0	1	0	0	1	0	0	0	0	0	0	0	0	0	0	0	0	0	0	0	0	0	0	0	0	0	0	0	0	0	0	0	0	0	0	0	0	0	0	0	0	0	0	0	0	0	0	0	0	0	0	0	0	0	0	0	0	0	0
R_Ulaancricetodon badamae	0	0	0	0	0	0	0	0	0	0	0	0	0	0	0	0	0	0	0	0	0	0	0	0	0	0	0	0	0	0	0	0	0	0	0	0	0	0	0	0	0	6	22	21	31	8	1	0	1	0	1	10	0	1	0	3	12	0	2	4
R_Selenomys mimicus	0	0	0	0	0	0	0	0	0	0	0	0	0	0	0	0	0	0	0	0	0	0	0	0	0	0	0	0	0	0	0	0	0	0	0	0	0	0	0	0	0	0	0	0	0	0	0	0	0	1	0	0	3	0	0	0	0	10	7	137
R_Cricetops dormitor	0	0	0	0	0	0	0	0	0	0	0	0	0	0	0	0	0	0	0	0	0	0	0	0	0	0	0	0	0	0	0	0	0	0	0	0	0	0	0	11	0	0	15	15	38	1	0	0	0	66	0	1	2	0	0	0	0	3	0	530
R_Cricetops minor	0	0	0	0	0	0	0	0	0	0	0	0	0	0	0	0	0	0	0	0	0	0	0	0	0	0	0	0	0	0	0	0	0	0	0	0	0	0	0	0	0	0	0	0	0	0	0	0	0	0	0	0	0	0	0	0	0	0	0	2
R_Edirnella /Witenia sp.	0	0	0	0	0	0	0	0	0	0	0	0	0	0	0	0	0	0	0	0	0	0	0	0	0	0	0	0	0	0	0	0	0	0	0	0	0	0	0	1	0	0	0	0	0	0	0	0	0	0	0	0	0	0	0	0	0	0	1	0
R_Eucricetodon caducus/ cf.caducus	0	0	0	0	0	0	0	0	0	0	0	0	0	0	0	0	0	0	0	0	0	0	0	0	0	0	0	0	0	0	0	0	0	0	0	0	0	0	0	0	0	6	2	1	3	6	0	3	2	4	2	1	0	0	0	6	5	9	7	49
R_Eucricetodon asiaticus / cf. asiat.	0	0	0	0	0	0	0	0	0	0	0	0	0	0	0	0	0	0	0	0	0	0	0	0	0	0	0	0	0	0	0	0	0	0	0	0	0	0	0	3	19	8	10	14	20	18	0	3	4	1	11	0	0	0	0	3	8	0	1	6
R_Eocricetodon meridionalis / cf. merid.	0	0	0	0	0	0	0	0	0	0	0	0	0	0	0	0	0	1	0	0	0	0	0	0	0	0	0	0	0	0	0	0	0	0	9	0	0	1	0	1	0	0	3	0	3	1	0	0	0	0	0	4	0	0	0	2	0	0	1	1
R_Eucricetodon occasionalis	0	0	0	0	0	0	0	0	0	0	0	0	0	0	0	0	0	0	0	0	0	0	0	0	0	0	0	0	0	0	0	0	0	0	0	0	0	0	0	0	0	0	2	0	0	5	0	0	0	0	0	0	0	0	0	0	0	0	0	0
R_Cricetidae indet.	0	0	0	0	0	0	0	0	0	0	0	0	0	0	0	0	0	0	0	0	0	0	0	0	0	0	0	0	0	0	0	0	0	0	0	0	0	1	2	0	0	0	0	0	0	0	0	0	0	3	1	0	0	0	0	1	0	0	0	0
R_Eucricetodon cf. bagus	0	0	0	0	0	0	0	0	0	0	0	0	0	0	0	0	0	1	0	0	0	0	0	0	0	1	1	0	0	0	3	0	0	0	87	0	14	8	92	1	0	0	0	0	0	3	0	1	0	0	3	0	0	0	0	0	0	0	0	0
R_Eymyarion tremulus	0	0	0	0	0	0	0	0	0	0	0	0	0	0	0	0	0	0	0	0	0	0	0	0	0	0	0	0	0	0	3	0	0	0	28	0	1	1	6	0	0	0	0	0	0	1	0	0	0	0	0	0	0	0	0	0	0	0	0	0
R_Eucricetodon jilantaniensis (large)	0	0	0	0	0	0	0	0	0	0	0	0	0	0	0	0	0	0	0	0	0	1	0	0	0	0	1	0	0	0	0	0	0	0	5	1	0	0	0	0	0	0	0	0	0	1	0	0	0	0	0	0	0	0	0	0	0	0	0	0
R_Eucricetodon sp.	0	0	0	0	0	0	0	0	0	0	0	0	0	0	0	0	0	0	0	0	0	0	0	0	0	0	0	0	0	0	0	0	0	0	42	0	1	0	4	0	0	0	2	1	0	0	0	1	0	0	0	0	0	0	0	0	0	0	0	0
R_Aralocricetodon schokensis	0	0	0	0	0	0	0	0	0	0	0	0	0	0	0	0	0	0	0	0	0	1	0	0	0	0	0	0	0	0	2	0	0	0	5	0	7	0	37	0	0	0	0	0	0	0	0	0	0	0	0	0	0	0	0	0	0	0	0	0
R_Argyromys sp.	0	0	0	0	0	0	0	0	0	0	0	0	0	0	0	0	0	0	0	0	0	0	0	0	0	1	0	0	0	0	0	0	0	0	16	0	0	0	0	0	0	0	0	0	0	0	0	0	0	0	0	0	0	0	0	0	0	0	0	0
R_Democricetodon sui	5	0	0	0	0	0	0	0	0	0	0	0	0	0	0	0	0	0	0	0	0	0	0	0	0	0	0	0	0	0	0	0	0	0	0	0	0	0	0	0	0	0	0	0	0	0	0	0	0	0	0	0	0	0	0	0	0	0	0	0
R_Primus sp.	0	0	0	1	0	0	0	0	0	0	0	0	0	0	0	0	0	0	0	0	0	0	0	0	0	0	0	0	0	0	0	0	0	0	0	0	0	0	0	0	0	0	0	0	0	0	0	0	0	0	0	0	0	0	0	0	0	0	0	0
Cr_Hyaenodon eminus	0	0	0	0	0	0	0	0	0	0	0	0	0	0	0	0	0	0	0	0	0	0	0	0	0	0	0	0	0	0	0	0	0	0	0	0	0	0	0	0	2	0	0	0	0	0	0	0	0	0	0	0	0	0	0	0	0	0	0	0
Cr_Hyaenodon pervagus	0	0	0	0	0	0	0	0	0	0	0	0	0	0	0	0	0	0	0	0	0	0	0	0	0	0	0	0	0	0	0	0	0	0	0	0	0	0	0	0	10	2	0	0	0	3	0	0	0	0	0	0	0	0	0	0	1	0	0	0
Cr_Hyaenodon incertus	0	0	0	0	0	0	0	0	0	0	0	0	0	0	0	0	0	0	0	0	0	0	0	0	0	0	0	0	0	0	0	0	0	0	0	0	0	0	0	0	2	0	0	0	0	1	0	0	0	0	0	0	0	0	0	0	0	0	0	1
Cr_Hyaenodon mongoliensis	0	0	0	0	0	0	0	0	0	0	0	0	0	0	0	0	0	0	0	0	0	0	0	0	0	0	0	0	0	0	0	0	0	0	0	0	0	0	0	0	0	0	0	0	0	0	0	0	0	0	0	0	0	0	0	1	0	0	0	0
Cr_Hyaenodon gigas	0	0	0	0	0	0	0	0	0	0	0	0	0	0	0	0	0	0	0	0	0	0	0	0	0	0	0	0	0	0	0	0	0	0	0	0	0	0	0	0	0	0	0	0	0	1	0	0	0	0	0	0	0	0	0	0	0	0	0	0
Cr_Hyaenodon sp.	0	0	0	0	0	0	0	0	0	0	0	0	0	0	0	0	2	0	0	0	0	0	0	0	0	0	0	0	10	0	1	0	0	5	0	0	0	0	0	0	6	9	0	0	0	0	0	0	2	0	0	0	0	0	0	0	0	0	0	0
Ca_Amphicynodon teilhardi	0	0	0	0	0	0	0	0	0	0	0	0	0	0	0	0	0	0	0	0	0	0	0	0	0	0	0	0	0	0	0	0	0	0	0	0	0	0	0	0	1	0	0	0	0	3	0	0	0	0	0	0	0	0	0	0	0	0	0	3
Ca_Amphicynodon sp.	0	0	0	0	0	0	0	0	0	0	0	0	0	0	0	0	0	0	0	0	0	0	0	0	0	0	0	0	0	0	0	0	0	0	0	0	0	0	1	0	2	0	0	0	0	0	0	0	0	0	0	0	0	0	0	0	0	0	0	0
Ca_Amphicticeps shackelfordi	0	0	0	0	0	0	0	0	0	0	0	0	0	0	0	0	0	0	0	0	0	0	0	0	0	0	0	0	0	0	1	0	0	0	0	0	0	0	0	0	0	0	0	0	0	1	0	0	0	0	0	0	0	0	0	0	0	0	0	0
Ca_Shandgolictis elegans	0	0	0	0	0	0	0	0	0	0	0	0	0	0	0	0	0	0	0	0	0	0	0	0	0	0	0	0	0	0	0	0	0	0	0	0	0	0	1	1	2	1	0	0	0	0	0	0	0	0	0	0	0	0	0	0	0	0	0	0
Ca_Asiavorator altidens	0	0	0	0	0	0	0	0	0	0	0	0	0	0	0	0	0	0	0	0	0	0	0	0	0	0	0	3	0	0	0	0	0	0	0	0	0	0	0	0	0	0	0	0	0	0	0	0	3	0	0	0	0	0	0	0	0	0	0	0
Ca_Asiavorator sp.	0	0	0	0	0	0	0	0	0	0	0	0	0	0	0	0	0	0	0	0	0	0	0	0	0	0	0	0	0	0	0	0	0	0	0	0	0	0	1	0	0	0	0	0	0	0	0	0	0	0	1	0	0	0	0	0	0	0	0	0
Ca_Nimravus mongoliensis	0	0	0	0	0	0	0	0	0	0	0	0	0	0	0	0	0	0	0	0	0	0	0	0	0	0	0	0	0	0	0	0	0	0	0	0	0	0	0	1	1	3	0	0	0	1	0	0	0	0	0	0	0	0	0	0	0	0	0	0
Ca_Palaeogale sectoria	0	0	0	0	0	0	0	0	0	0	0	0	0	0	0	0	0	0	0	0	2	0	0	0	0	0	0	0	0	0	0	0	0	0	0	0	0	0	1	0	0	6	4	1	0	6	0	4	0	0	0	0	0	0	0	0	0	0	0	0
Carnivora indet.	0	0	0	0	0	6	0	3	2	0	0	0	0	0	0	0	0	0	0	0	0	0	0	0	0	0	0	0	0	0	0	0	0	0	0	0	0	0	0	0	0	3	0	1	0	2	0	0	0	0	0	0	0	0	0	0	0	0	0	1
Di_Ergilictis sp.	0	0	0	0	0	0	0	0	0	0	0	0	0	0	0	0	0	0	0	0	0	0	0	0	0	0	0	0	0	0	0	0	0	0	0	0	0	0	0	0	0	0	1	0	0	0	0	0	0	0	0	0	0	0	0	0	0	0	0	0
Di_Didymoconus colgatei	0	0	0	0	0	0	0	0	0	0	0	0	0	0	0	0	0	0	0	0	0	0	0	0	0	0	0	0	0	0	0	0	0	0	0	0	0	0	3	0	0	1	0	0	0	0	0	0	0	0	0	0	0	0	0	0	0	0	0	1
Di_Didymoconus berkey	0	0	0	0	0	0	0	0	0	0	0	0	0	0	0	0	0	0	0	0	2	0	4	0	0	0	0	0	0	0	0	0	0	0	0	0	0	0	0	1	0	0	0	0	0	0	0	0	0	0	0	0	0	0	0	0	0	0	0	0
Di_Didymoconus sp.	0	0	0	0	0	0	0	0	0	0	0	0	0	0	0	0	2	0	0	3	0	0	0	0	1	0	0	0	2	2	0	0	0	0	0	0	0	0	0	0	0	0	0	0	0	0	0	0	0	0	0	0	0	0	0	0	0	0	0	0
A_Praetragulus gobiae	0	0	0	0	0	0	0	0	0	0	0	0	0	0	0	0	0	0	0	0	0	0	0	0	0	0	0	0	0	0	0	0	0	0	0	0	0	0	0	0	0	0	0	0	1	0	0	0	0	0	0	0	0	0	0	0	0	0	0	2

A_Miomeryx sp.	0	0	0	0	0	0	0	0	0	0	0	0	0	0	0	0	0	0	0	0	0	0	0	0	0	0	0	0	0	0	0	0	0	0	0	0	0	0	0	0	0	0	1	1	0	2	0	0	0	0	0	0	0	0	0	0	0	0	0	1
A_Gobimeryx dubius	0	0	0	0	0	0	0	0	0	0	0	0	0	0	0	0	0	0	0	0	0	0	0	0	0	0	0	0	0	0	0	0	0	0	0	0	0	0	0	0	0	0	0	0	0	0	0	0	0	0	0	0	0	0	0	0	0	0	0	8
A_Gobimeryx sp.	0	0	0	0	0	0	0	0	0	0	0	0	0	0	0	0	0	0	0	0	0	0	0	0	0	0	0	0	0	0	0	0	0	0	0	0	0	0	0	0	0	3	1	0	0	0	1	0	0	0	0	0	0	1	0	0	0	0	0	0
A_Pseudomeryx gobiensis	0	0	0	0	0	0	0	0	0	0	0	0	0	0	0	0	0	0	0	0	0	0	0	0	0	0	0	0	0	0	0	0	0	0	0	0	0	0	0	0	0	0	0	0	0	0	0	0	0	1	0	0	0	0	0	0	0	0	0	10
A_Pseudomeryx sp.	0	0	0	0	0	0	0	0	0	0	0	0	0	0	0	0	0	0	0	0	0	0	0	0	0	0	0	0	0	0	0	0	0	0	0	0	0	0	0	1	0	0	1	0	1	1	0	0	0	0	0	0	0	0	0	0	0	0	0	1
A_Prodremotherium sp.	0	0	0	0	0	0	0	0	0	0	0	0	0	0	0	0	0	0	0	0	0	0	0	0	0	0	0	0	0	0	0	0	0	0	0	0	0	0	3	0	0	0	0	0	0	3	0	0	0	0	0	0	0	0	0	0	0	0	0	0
A_Pseudogelocus mongolicus	0	0	0	0	0	0	0	0	0	0	0	0	0	0	0	0	0	0	0	0	0	0	0	0	0	0	0	0	0	0	0	0	0	0	0	0	0	0	0	0	0	0	4	0	0	1	0	0	1	0	0	0	0	0	0	0	0	0	0	3
A_Paragelocus aff. scotti	0	0	0	0	0	0	0	0	0	0	0	0	0	0	0	0	0	0	0	0	0	0	0	0	0	0	0	0	0	0	0	0	0	0	0	1	0	0	0	3	0	0	0	0	0	0	0	0	0	0	0	0	0	0	0	0	0	0	0	0
A_Lophiomeryx angarae	0	0	0	0	0	0	0	0	0	0	0	0	0	0	0	0	0	0	0	0	0	0	0	0	0	0	0	0	0	0	0	0	0	0	0	0	0	0	0	0	0	0	0	0	0	0	0	0	0	0	0	0	0	0	0	1	0	0	0	1
A_Lophiomeryx sp.	0	0	0	0	0	0	0	0	0	0	0	0	0	0	0	0	0	0	0	0	0	0	0	0	0	0	0	0	0	0	0	0	0	0	0	0	0	0	0	0	0	0	0	0	0	0	0	0	0	0	0	0	0	0	0	0	0	0	0	1
A_Eumeryx culminis	0	0	0	0	0	0	0	0	0	0	0	0	0	0	0	0	0	0	0	0	0	0	0	0	0	0	0	0	0	0	0	0	0	0	0	0	0	0	0	0	0	0	0	0	0	0	0	0	0	0	0	0	0	0	0	1	1	0	0	0
A_Eumeryx imbellis	0	0	0	0	0	0	0	0	0	0	0	0	0	0	0	0	0	0	0	0	0	0	0	0	0	0	0	0	0	0	0	0	0	0	0	0	0	0	0	0	0	2	0	0	0	2	0	0	0	0	0	0	0	0	0	0	0	0	0	0
A_Eumeryx sp.	0	0	0	0	0	0	0	0	0	0	0	0	0	0	0	0	0	0	0	0	0	0	0	0	0	0	0	0	0	0	0	0	0	0	1	0	0	0	0	0	0	0	0	0	1	6	0	0	0	1	0	0	0	0	0	0	0	0	0	0
A_Amphitragulus sp.	0	0	0	0	0	0	0	0	0	0	0	0	0	0	0	0	0	0	0	0	0	0	0	0	0	1	0	0	0	0	0	0	0	0	0	0	0	0	0	0	0	0	0	0	0	0	0	0	0	0	0	0	0	0	0	0	0	0	0	0
A_Dremotherium cf. guthi	0	0	0	0	0	0	0	0	0	0	0	0	0	0	0	0	0	0	0	0	0	0	0	0	0	0	0	0	0	0	0	0	0	0	0	0	0	0	1	0	0	0	0	0	0	0	0	0	0	0	0	0	0	0	0	0	0	0	0	0
A_Palaeohypsodontus sp.	0	0	0	0	0	0	0	0	0	0	0	0	0	0	0	0	0	0	0	0	0	0	0	0	0	0	0	0	0	0	2	0	0	0	1	0	0	0	0	0	0	0	0	0	0	0	0	0	0	0	0	0	0	0	0	0	0	0	0	0
A_Bovidae gen. 1	0	0	0	0	0	0	0	0	0	0	0	0	0	0	0	0	0	0	0	0	0	0	0	0	0	0	0	0	0	0	0	0	0	0	0	1	0	0	0	0	0	0	0	0	0	0	0	0	0	0	0	0	0	0	0	0	0	0	0	0
A_Bovidae gen. 2	0	0	0	0	0	0	0	0	0	0	0	0	0	0	0	0	0	0	0	0	0	0	0	0	0	0	0	0	0	0	1	0	0	0	0	0	0	0	0	0	0	0	0	0	0	0	0	0	0	0	0	0	0	0	0	0	0	0	0	0
A_Gobiocerus sp.	0	0	0	0	0	0	0	0	0	0	0	0	0	0	0	0	0	0	0	0	0	0	0	0	0	1	0	0	0	0	0	0	0	0	0	0	0	0	0	0	0	0	0	0	0	0	0	0	0	0	0	0	0	0	0	0	0	0	0	0
A_Ruminantia indet.	6	0	0	0	0	6	0	0	5	0	0	0	0	0	3	0	1	0	0	1	0	0	1	0	3	0	0	0	0	15	0	0	0	2	0	1	0	0	18	0	5	23	2	2	0	2	0	3	1	0	1	7	0	0	0	0	1	0	0	46
P_Rhinocerotidae indet.	4	0	0	1	0	0	0	0	2	0	0	6	0	0	0	0	0	0	0	0	0	0	0	0	0	0	0	0	0	0	0	0	0	0	0	0	0	0	0	0	0	0	0	0	0	0	0	0	0	0	0	0	0	0	0	0	0	0	0	0
P_Paraceratherium sp.	0	0	0	0	0	0	0	0	0	0	0	0	0	0	0	0	0	0	0	0	1	0	0	0	0	0	0	0	0	0	0	0	0	0	0	0	0	0	0	0	0	0	0	0	0	0	0	0	0	0	0	0	0	0	0	0	0	0	0	0
P_Benaratherium sp.	0	0	0	0	0	0	0	0	0	0	0	0	0	0	0	0	0	0	0	0	3	0	0	0	0	0	0	0	0	0	0	0	0	0	0	0	0	0	0	0	0	0	0	0	0	0	0	0	0	0	0	0	0	0	0	0	0	0	0	0
P_Aceratherium pauliacense	0	0	0	0	0	0	0	0	0	0	0	0	0	0	0	0	0	0	0	0	1	0	0	0	0	0	0	0	0	0	0	0	0	0	0	0	0	0	0	0	0	0	0	0	0	0	0	0	0	0	0	0	0	0	0	0	0	0	0	0
P_Hoploaceratherium gobiense	5	0	0	0	5	13	0	0	0	0	0	0	0	0	0	0	0	0	0	0	0	0	0	0	0	0	0	0	0	0	0	0	0	0	0	0	0	0	0	0	0	0	0	0	0	0	0	0	0	0	0	0	0	0	0	0	0	0	0	0
P_Elasmotheriini indet.	0	0	0	0	0	0	0	0	0	0	0	0	0	0	0	0	0	0	0	0	1	0	0	0	0	0	0	0	0	0	0	0	0	0	0	0	0	0	0	0	0	0	0	0	0	0	0	0	0	0	0	0	0	0	0	0	0	0	0	0
P_Caementodon sp.	1	0	0	0	0	1	0	0	0	0	0	0	0	0	0	0	0	0	0	0	0	0	0	0	0	0	0	0	0	0	0	0	0	0	0	0	0	0	0	0	0	0	0	0	0	0	0	0	0	0	0	0	0	0	0	0	0	0	0	0

Supplementary Table 2: $\delta^{13}C$ values of paleosol carbonate.

AGE	BIOZONE	SAMPLE	AGE	δ13C
21,6	biozone D	HTE/13a carb	21,6	-8,1
21,7	biozone D	HTE/12 carb	21,7	-4,4
21,9	biozone D	HTE/11b carb	21,9	-5,4
22,1	biozone D	HTE/11a carb	22,1	-7,3
22,3	biozone D	HTE/9b carb	22,3	-5,1
22,5	biozone D	HTE/9a carb	22,5	-6,3
22,6	biozone D	HTE/8 carb	22,6	-6,1
22,8	biozone D	HTE/7b carb	22,8	-5,7
23	biozone D	HTE/7a carb	23	-6,4
23,1	biozone C1-D	HTE/6 carb	23,1	-6,3
23,2	biozone C1-D	HTE/5b carb	23,2	-8,9
23,7	biozone C1-D	HTE/4b carb	23,7	-8,96
23,8	biozone C1-D	HTE/4a carb	23,8	-6,30
24,12	biozone C1-D	TGR-C/19b	24,12	-3,78
24,24	biozone C1-D	TGR-C/19a	24,24	-4,77
24,36	biozone C1	TGR-C/18	24,36	-4,67
24,5	biozone C1	TGR-C/17b	24,5	-4,98
24,7	biozone C1	TGR-C/17a	24,7	-4,51
24,9	biozone C1	TGR-C/16c	24,9	-4,94
25,1	biozone C1	TGR-C/15	25,1	-6,09
25,2	biozone C1	TGR-C/14b	25,2	-6,09
25,3	biozone C1	TGR-C/13b	25,3	-5,7
25,4	biozone C1	TGR-C/12	25,4	-5,4
25,5	biozone C1	TGR-C/11a	25,5	-6,8
25,6	biozone C1	TGR-C/10b	25,6	-5,9
25,7	biozone C	TGR-C/10a	25,7	-5,7
25,8	biozone C	TGR-C/9	25,8	-5,8
25,9	biozone C	TGR-C/8b	25,9	-5,6
26	biozone C	TGR-C/8a	26	-5,8
26,1	biozone C	TGR-C/7c	26,1	-5,5
26,2	biozone C	TGR-C/7b	26,2	-5,4
26,3	biozone C	TGR-C/7a	26,3	-5,9
26,4	biozone C	TGR-C/6	26,4	-5,9
26,5	biozone C	TGR-C/5	26,5	-5,4
26,7	biozone C	TGR-C/4b	26,7	-5,6
26,8	biozone C	TGR-C/4a	26,8	-5,6
26,9	biozone C	TGR-C/3A	26,9	-6,1
27	biozone C	TGR-C/3c	27	-5,1
27,1	biozone C	TGR-C/3b	27,1	-5,5
27,2	biozone C	TGR-C/3a	27,2	-5,3
27,3	biozone C	TGR-C/2	27,3	-5,9
27,4	biozone C	TGR-C/1	27,4	-5,9
28	biozone B	SHG-D/25	28	-5,4
28,1	biozone B	SHG-D/24	28,1	-5,2
28,2	biozone B	SHG-D/23j	28,2	-5,5
28,3	biozone B	SHG-D/23i	28,3	-5,3
28,4	biozone B	SHG-D/23h	28,4	-5,4
28,5	biozone B	SHG-D/23g	28,5	-5,0
28,6	biozone B	SHG-D/23f	28,6	-5,2
28,7	biozone B	SHG-D/23e	28,7	-5,3
28,8	biozone B	SHG-D/23d	28,8	-5,4
28,9	biozone B	SHG-D/23c	28,9	-4,6
29	biozone B	SHG-D/23b	29	-5,4
29,1	biozone B	SHG-D/23a	29,1	-5,0
29,2	biozone B	SHG-D/22	29,2	-5,2
29,3	biozone B	SHG-D/21	29,3	-5,1
29,4	biozone B	SHG-D/20b	29,4	-5,5
29,5	biozone B	SHG-D/20a	29,5	-5,8
29,6	biozone B	SHG-D/19	29,6	-4,9
29,7	biozone B	SHG-D/18	29,7	-5,3

29,8	biozone B	SHG-D/17	29,8	-5,1
29,9	biozone B	SHG-D/16c	29,9	-5,6
30	biozone B	SHG-D/16b	30	-5,6
30,15	biozone B	SHG-D/16a	30,15	-5,5
30,3	biozone B	SHG-D/15	30,3	-5,1
30,45	biozone B	SHG-D/14b	30,45	-5,9
30,6	biozone B	SHG-D/14a	30,6	-5,6
30,75	biozone B	SHG-D/13	30,75	-5,3
30,9	biozone B	SHG-D/6	30,9	-7,3
31,1	biozone B	SHG-D/2	31,1	-7,6
31,7	biozone A	TGR-AB/33b	31,7	-8,3
31,8	biozone A	TGR-AB/33a	31,8	-7,2
31,9	biozone A	TGR-AB/32l	31,9	-7,5
32	biozone A	TGR-AB/32k	32	-7,0
32,1	biozone A	TGR-AB/32j	32,1	-7,0
32,2	biozone A	TGR-AB/32i	32,2	-6,8
32,3	biozone A	TGR-AB/32h	32,3	-6,9
32,4	biozone A	TGR-AB/32g	32,4	-6,6
32,5	biozone A	TGR-AB/32f	32,5	-6,8
32,6	biozone A	TGR-AB/32e	32,6	-6,8
32,7	biozone A	TGR-AB/32d	32,7	-6,7
32,9	biozone A	TGR-AB/32c	32,9	-6,4
33,1	biozone A	TGR-AB/32b	33,1	-7,2
33,3	biozone A	TGR-AB/32a	33,3	-7,0

Supplementary Table 3: Magnetic susceptibility in SI units (10^{-6})

AGE	BIOZONE	SECTION	UNIT	MS
21,40	biozone D	HTE	16	11
21,41	biozone D	HTE	16	15
21,42	biozone D	HTE	15	120
21,43	biozone D	HTE	15	140
21,44	biozone D	HTE	15	110
21,45	biozone D	HTE	15	144
21,46	biozone D	HTE	15	136
21,47	biozone D	HTE	15	93
21,48	biozone D	HTE	15	113
21,49	biozone D	HTE	14	75
21,50	biozone D	HTE	14	107
21,51	biozone D	HTE	14	152
21,52	biozone D	HTE	14	115
21,53	biozone D	HTE	14	139
21,54	biozone D	HTE	14	107
21,55	biozone D	HTE	14	153
21,56	biozone D	HTE	14	128
21,57	biozone D	HTE	14	109
21,58	biozone D	HTE	13	133
21,59	biozone D	HTE	13	102
21,60	biozone D	HTE	13	144
21,61	biozone D	HTE	13	116
21,62	biozone D	HTE	13	136
21,63	biozone D	HTE	13	112
21,64	biozone D	HTE	13	105
21,65	biozone D	HTE	13	81
21,66	biozone D	HTE	13	125
21,67	biozone D	HTE	13	99
21,68	biozone D	HTE	13	91
21,69	biozone D	HTE	13	111
21,70	biozone D	HTE	13	78
21,71	biozone D	HTE	13	130
21,72	biozone D	HTE	13	147
21,73	biozone D	HTE	13	109
21,74	biozone D	HTE	13	106
21,75	biozone D	HTE	13	121
21,76	biozone D	HTE	13	111
21,77	biozone D	HTE	13	113
21,78	biozone D	HTE	13	146
21,79	biozone D	HTE	13	131
21,80	biozone D	HTE	13	152
21,81	biozone D	HTE	13	147
21,82	biozone D	HTE	13	156
21,83	biozone D	HTE	13	138
21,84	biozone D	HTE	13	128
21,85	biozone D	HTE	13	197
21,86	biozone D	HTE	13	187
21,87	biozone D	HTE	13	240
21,88	biozone D	HTE	13	262
21,89	biozone D	HTE	13	302
21,90	biozone D	HTE	13	226
21,91	biozone D	HTE	13	217
21,92	biozone D	HTE	13	206
21,93	biozone D	HTE	13	156
21,94	biozone D	HTE	13	232
21,95	biozone D	HTE	13	115
21,96	biozone D	HTE	13	187
21,97	biozone D	HTE	13	176
21,98	biozone D	HTE	13	129
21,99	biozone D	HTE	12	210

22,00	biozone D	HTE	12	266
22,01	biozone D	HTE	12	169
22,02	biozone D	HTE	12	268
22,03	biozone D	HTE	12	300
22,04	biozone D	HTE	12	190
22,05	biozone D	HTE	12	135
22,06	biozone D	HTE	12	173
22,07	biozone D	HTE	12	110
22,08	biozone D	HTE	12	183
22,09	biozone D	HTE	12	168
22,10	biozone D	HTE	12	165
22,11	biozone D	HTE	12	122
22,12	biozone D	HTE	12	81
22,13	biozone D	HTE	12	121
22,14	biozone D	HTE	12	145
22,15	biozone D	HTE	12	157
22,16	biozone D	HTE	12	193
22,17	biozone D	HTE	12	172
22,18	biozone D	HTE	12	197
22,19	biozone D	HTE	12	196
22,20	biozone D	HTE	12	141
22,21	biozone D	HTE	12	76
22,22	biozone D	HTE	12	183
22,23	biozone D	HTE	12	83
22,24	biozone D	HTE	11	140
22,25	biozone D	HTE	11	204
22,26	biozone D	HTE	11	141
22,27	biozone D	HTE	11	151
22,28	biozone D	HTE	11	131
22,29	biozone D	HTE	11	104
22,30	biozone D	HTE	11	150
22,31	biozone D	HTE	11	157
22,32	biozone D	HTE	11	96
22,33	biozone D	HTE	11	131
22,34	biozone D	HTE	11	170
22,35	biozone D	HTE	11	172
22,36	biozone D	HTE	11	150
22,37	biozone D	HTE	11	119
22,38	biozone D	HTE	11	189
22,39	biozone D	HTE	11	184
22,40	biozone D	HTE	11	150
22,41	biozone D	HTE	11	214
22,42	biozone D	HTE	11	202
22,43	biozone D	HTE	11	199
22,44	biozone D	HTE	11	330
22,45	biozone D	HTE	11	98
22,46	biozone D	HTE	11	89
22,47	biozone D	HTE	11	143
22,48	biozone D	HTE	11	80
22,49	biozone D	HTE	11	106
22,50	biozone D	HTE	11	90
22,51	biozone D	HTE	11	69
22,52	biozone D	HTE	10	159
22,53	biozone D	HTE	10	129
22,54	biozone D	HTE	10	165
22,55	biozone D	HTE	10	170
22,56	biozone D	HTE	10	183
22,57	biozone D	HTE	9	265
22,58	biozone D	HTE	9	195
22,59	biozone D	HTE	9	269
22,60	biozone D	HTE	9	394
22,61	biozone D	HTE	9	219
22,62	biozone D	HTE	9	364

22,63	biozone D	HTE	9	190
22,64	biozone D	HTE	9	340
22,65	biozone D	HTE	9	169
22,67	biozone D	HTE	8	445
22,68	biozone D	HTE	8	149
22,69	biozone D	HTE	8	112
22,70	biozone D	HTE	8	206
22,74	biozone D	HTE	7	74
22,75	biozone D	HTE	7	65
22,76	biozone D	HTE	7	36
22,77	biozone D	HTE	7	59
22,78	biozone D	HTE	7	96
22,79	biozone D	HTE	7	89
22,80	biozone D	HTE	7	84
22,81	biozone D	HTE	7	125
22,82	biozone D	HTE	7	198
22,83	biozone D	HTE	7	99
22,84	biozone D	HTE	7	115
22,85	biozone D	HTE	7	173
22,86	biozone D	HTE	7	230
22,87	biozone D	HTE	7	131
22,88	biozone D	HTE	7	134
22,89	biozone D	HTE	7	183
22,90	biozone D	HTE	7	175
22,91	biozone D	HTE	7	135
22,92	biozone D	HTE	7	132
22,93	biozone D	HTE	7	153
22,94	biozone D	HTE	7	162
22,95	biozone D	HTE	7	167
23,00	biozone D	HTE	7	127
23,20	biozone C1-D	HTE	6	117
23,21	biozone C1-D	HTE	6	223
23,22	biozone C1-D	HTE	6	143
23,23	biozone C1-D	HTE	6	222
23,24	biozone C1-D	HTE	6	215
23,26	biozone C1-D	HTE	6	152
23,27	biozone C1-D	HTE	6	146
23,28	biozone C1-D	HTE	6	267
23,29	biozone C1-D	HTE	6	111
23,30	biozone C1-D	HTE	6	201
23,31	biozone C1-D	HTE	5	108
23,32	biozone C1-D	HTE	5	119
23,33	biozone C1-D	HTE	5	85
23,34	biozone C1-D	HTE	5	125
23,35	biozone C1-D	HTE	5	111
23,37	biozone C1-D	HTE	5	144
23,38	biozone C1-D	HTE	5	104
23,39	biozone C1-D	HTE	5	120
23,40	biozone C1-D	HTE	5	151
23,41	biozone C1-D	HTE	5	85
23,42	biozone C1-D	HTE	5	124
23,43	biozone C1-D	HTE	5	58
23,44	biozone C1-D	HTE	5	127
23,45	biozone C1-D	HTE	4	72
23,46	biozone C1-D	HTE	4	105
23,48	biozone C1-D	HTE	4	84
23,49	biozone C1-D	HTE	4	141
23,50	biozone C1-D	HTE	4	178
23,51	biozone C1-D	HTE	4	158
23,52	biozone C1-D	HTE	4	94
23,53	biozone C1-D	HTE	4	64
23,54	biozone C1-D	HTE	4	105
23,55	biozone C1-D	HTE	4	110

23,56	biozone C1-D	HTE	4	149
23,57	biozone C1-D	HTE	4	138
23,59	biozone C1-D	HTE	4	156
23,60	biozone C1-D	HTE	4	152
23,61	biozone C1-D	HTE	4	195
23,62	biozone C1-D	HTE	4	140
23,63	biozone C1-D	HTE	4	163
23,64	biozone C1-D	HTE	4	118
23,65	biozone C1-D	HTE	4	150
23,66	biozone C1-D	HTE	3	287
23,67	biozone C1-D	HTE	3	197
23,68	biozone C1-D	HTE	3	171
23,70	biozone C1-D	HTE	3	186
23,71	biozone C1-D	HTE	3	173
23,72	biozone C1-D	HTE	3	132
23,73	biozone C1-D	HTE	3	103
23,74	biozone C1-D	HTE	3	125
23,75	biozone C1-D	HTE	3	104
23,76	biozone C1-D	HTE	3	116
23,77	biozone C1-D	HTE	3	100
24,00	biozone C1	TAT-E	31	108
24,00	biozone C1	TAT-E	31	147
24,01	biozone C1	TAT-E	31	104
24,01	biozone C1	TAT-E	31	181
24,01	biozone C1	TAT-E	30	118
24,01	biozone C1	TAT-E	30	59
24,02	biozone C1	TAT-E	30	140
24,02	biozone C1	TAT-E	30	93
24,02	biozone C1	TAT-E	30	104
24,02	biozone C1	TAT-E	30	134
24,03	biozone C1	TAT-E	30	137
24,03	biozone C1	TAT-E	30	270
24,03	biozone C1	TAT-E	30	83
24,03	biozone C1	TAT-E	30	89
24,04	biozone C1	TAT-E	30	122
24,04	biozone C1	TAT-E	30	87
24,04	biozone C1	TAT-E	30	133
24,04	biozone C1	TAT-E	30	238
24,05	biozone C1	TAT-E	30	164
24,05	biozone C1	TAT-E	30	211
24,05	biozone C1	TAT-E	30	124
24,05	biozone C1	TAT-E	30	122
24,06	biozone C1	TAT-E	30	141
24,06	biozone C1	TAT-E	30	157
24,06	biozone C1	TAT-E	30	114
24,07	biozone C1	TAT-E	30	123
24,07	biozone C1	TAT-E	30	178
24,07	biozone C1	TAT-E	30	136
24,07	biozone C1	TAT-E	29	219
24,08	biozone C1	TAT-E	29	220
24,08	biozone C1	TAT-E	29	189
24,08	biozone C1	TAT-E	29	112
24,08	biozone C1	TAT-E	29	112
24,09	biozone C1	TAT-E	29	222
24,09	biozone C1	TAT-E	29	258
24,09	biozone C1	TAT-E	29	178
24,09	biozone C1	TAT-E	29	252
24,10	biozone C1	TAT-E	29	208
24,10	biozone C1	TAT-E	29	234
24,10	biozone C1	TAT-E	29	172
24,10	biozone C1	TAT-E	29	184
24,11	biozone C1	TAT-E	29	211
24,11	biozone C1	TAT-E	29	136

24,11	biozone C1	TAT-E	29	148
24,11	biozone C1	TAT-E	29	230
24,12	biozone C1	TAT-E	29	150
24,12	biozone C1	TAT-E	29	183
24,12	biozone C1	TAT-E	29	199
24,12	biozone C1	TAT-E	29	158
24,13	biozone C1	TAT-E	29	197
24,13	biozone C1	TAT-E	29	185
24,13	biozone C1	TAT-E	29	141
24,14	biozone C1	TAT-E	29	132
24,14	biozone C1	TAT-E	28	171
24,14	biozone C1	TAT-E	28	212
24,14	biozone C1	TAT-E	28	165
24,15	biozone C1	TAT-E	28	135
24,15	biozone C1	TAT-E	28	194
24,15	biozone C1	TAT-E	27	106
24,15	biozone C1	TAT-E	27	221
24,16	biozone C1	TAT-E	27	188
24,16	biozone C1	TAT-E	27	195
24,16	biozone C1	TAT-E	27	160
24,16	biozone C1	TAT-E	27	127
24,17	biozone C1	TAT-E	27	161
24,17	biozone C1	TAT-E	27	273
24,17	biozone C1	TAT-E	27	212
24,17	biozone C1	TAT-E	27	124
24,18	biozone C1	TAT-E	27	141
24,18	biozone C1	TAT-E	27	224
24,18	biozone C1	TAT-E	27	321
24,18	biozone C1	TAT-E	27	227
24,19	biozone C1	TAT-E	27	308
24,19	biozone C1	TAT-E	27	192
24,19	biozone C1	TAT-E	27	170
24,20	biozone C1	TAT-E	27	180
24,20	biozone C1	TAT-E	27	196
24,20	biozone C1	TAT-E	27	151
24,20	biozone C1	TAT-E	27	221
24,21	biozone C1	TAT-E	27	159
24,21	biozone C1	TAT-E	27	127
24,21	biozone C1	TAT-E	27	279
24,21	biozone C1	TAT-E	27	229
24,22	biozone C1	TAT-E	27	204
24,22	biozone C1	TAT-E	27	210
24,22	biozone C1	TAT-E	26	70
24,22	biozone C1	TAT-E	26	67
24,23	biozone C1	TAT-E	26	144
24,23	biozone C1	TAT-E	26	53
24,23	biozone C1	TAT-E	26	72
24,23	biozone C1	TAT-E	26	73
24,24	biozone C1	TAT-E	26	199
24,24	biozone C1	TAT-E	26	204
24,24	biozone C1	TAT-E	26	239
24,24	biozone C1	TAT-E	26	231
24,25	biozone C1	TAT-E	26	184
24,25	biozone C1	TAT-E	25	124
24,25	biozone C1	TAT-E	25	101
24,25	biozone C1	TAT-E	25	95
24,26	biozone C1	TAT-E	25	125
24,26	biozone C1	TAT-E	25	94
24,26	biozone C1	TAT-E	25	156
24,27	biozone C1	TAT-E	25	136
24,27	biozone C1	TAT-E	25	99
24,27	biozone C1	TAT-E	25	138
24,27	biozone C1	TAT-E	25	136

24,28	biozone C1	TAT-E	25	156
24,28	biozone C1	TAT-E	25	155
24,28	biozone C1	TAT-E	25	133
24,28	biozone C1	TAT-E	25	147
24,29	biozone C1	TAT-E	25	135
24,29	biozone C1	TAT-E	25	114
24,29	biozone C1	TAT-E	25	127
24,29	biozone C1	TAT-E	25	118
24,30	biozone C1	TAT-E	25	120
24,30	biozone C1	TAT-E	24	139
24,30	biozone C1	TAT-E	24	143
24,30	biozone C1	TAT-E	24	132
24,31	biozone C1	TAT-E	24	137
24,31	biozone C1	TAT-E	24	118
24,31	biozone C1	TAT-E	24	66
24,31	biozone C1	TAT-E	24	118
24,32	biozone C1	TAT-E	24	111
24,32	biozone C1	TAT-E	24	119
24,32	biozone C1	TAT-E	24	140
24,33	biozone C1	TAT-E	24	149
24,33	biozone C1	TAT-E	24	128
24,33	biozone C1	TAT-E	24	103
24,33	biozone C1	TAT-E	24	133
24,34	biozone C1	TAT-E	24	124
24,34	biozone C1	TAT-E	24	145
24,34	biozone C1	TAT-E	24	131
24,34	biozone C1	TAT-E	24	103
24,35	biozone C1	TAT-E	24	144
24,35	biozone C1	TAT-E	24	137
24,35	biozone C1	TAT-E	24	126
24,35	biozone C1	TAT-E	24	106
24,36	biozone C1	TAT-E	24	147
24,36	biozone C1	TAT-E	24	155
24,36	biozone C1	TAT-E	24	132
24,36	biozone C1	TAT-E	24	119
24,37	biozone C1	TAT-E	24	137
24,37	biozone C1	TAT-E	24	144
24,37	biozone C1	TAT-E	24	108
24,37	biozone C1	TAT-E	23	55
24,38	biozone C1	TAT-E	23	77
24,38	biozone C1	TAT-E	23	94
24,38	biozone C1	TAT-E	23	56
24,38	biozone C1	TAT-E	23	55
24,39	biozone C1	TAT-E	23	52
24,39	biozone C1	TAT-E	23	65
24,40	biozone C1	TAT-E	23	105
24,45	biozone C1	TGR-C	18	205
24,46	biozone C1	TGR-C	18	134
24,47	biozone C1	TGR-C	18	114
24,47	biozone C1	TGR-C	18	169
24,48	biozone C1	TGR-C	18	215
24,48	biozone C1	TGR-C	18	122
24,49	biozone C1	TGR-C	18	78
24,50	biozone C1	TGR-C	18	86
24,50	biozone C1	TGR-C	18	97
24,51	biozone C1	TGR-C	18	183
24,51	biozone C1	TGR-C	18	118
24,52	biozone C1	TGR-C	18	164
24,53	biozone C1	TGR-C	18	147
24,53	biozone C1	TGR-C	17	75
24,54	biozone C1	TGR-C	17	123
24,55	biozone C1	TGR-C	17	113
24,55	biozone C1	TGR-C	17	61

24,56	biozone C1	TGR-C	17	70
24,56	biozone C1	TGR-C	17	57
24,57	biozone C1	TGR-C	17	81
24,58	biozone C1	TGR-C	17	106
24,58	biozone C1	TGR-C	17	103
24,59	biozone C1	TGR-C	17	82
24,59	biozone C1	TGR-C	17	205
24,60	biozone C1	TGR-C	17	121
24,61	biozone C1	TGR-C	16	197
24,61	biozone C1	TGR-C	16	98
24,62	biozone C1	TGR-C	16	166
24,62	biozone C1	TGR-C	16	104
24,63	biozone C1	TGR-C	16	143
24,64	biozone C1	TGR-C	16	115
24,64	biozone C1	TGR-C	16	139
24,65	biozone C1	TGR-C	16	111
24,66	biozone C1	TGR-C	16	105
24,66	biozone C1	TGR-C	16	144
24,67	biozone C1	TGR-C	16	181
24,67	biozone C1	TGR-C	16	152
24,68	biozone C1	TGR-C	16	150
24,69	biozone C1	TGR-C	16	145
24,69	biozone C1	TGR-C	16	196
24,70	biozone C1	TGR-C	16	158
24,70	biozone C1	TGR-C	16	200
24,71	biozone C1	TGR-C	16	154
24,72	biozone C1	TGR-C	16	197
24,72	biozone C1	TGR-C	16	148
24,73	biozone C1	TGR-C	16	179
24,73	biozone C1	TGR-C	16	150
24,74	biozone C1	TGR-C	16	239
24,75	biozone C1	TGR-C	16	189
24,75	biozone C1	TGR-C	16	130
24,76	biozone C1	TGR-C	16	162
24,76	biozone C1	TGR-C	16	160
24,77	biozone C1	TGR-C	16	163
24,78	biozone C1	TGR-C	16	214
24,78	biozone C1	TGR-C	16	112
24,79	biozone C1	TGR-C	16	111
24,80	biozone C1	TGR-C	16	108
24,80	biozone C1	TGR-C	15	102
24,81	biozone C1	TGR-C	15	113
24,81	biozone C1	TGR-C	15	164
24,82	biozone C1	TGR-C	15	134
24,83	biozone C1	TGR-C	15	124
24,83	biozone C1	TGR-C	15	128
24,84	biozone C1	TGR-C	15	154
24,84	biozone C1	TGR-C	15	107
24,85	biozone C1	TGR-C	15	140
24,86	biozone C1	TGR-C	15	97
24,86	biozone C1	TGR-C	14	155
24,87	biozone C1	TGR-C	14	164
24,87	biozone C1	TGR-C	14	153
24,88	biozone C1	TGR-C	14	116
24,89	biozone C1	TGR-C	14	148
24,89	biozone C1	TGR-C	14	110
24,90	biozone C1	TGR-C	14	153
24,91	biozone C1	TGR-C	14	177
24,91	biozone C1	TGR-C	14	112
24,92	biozone C1	TGR-C	14	144
24,92	biozone C1	TGR-C	14	152
24,93	biozone C1	TGR-C	14	144
24,94	biozone C1	TGR-C	14	137

24,94	biozone C1	TGR-C	14	116
24,95	biozone C1	TGR-C	14	92
24,95	biozone C1	TGR-C	14	64
24,96	biozone C1	TGR-C	14	97
24,97	biozone C1	TGR-C	14	73
24,97	biozone C1	TGR-C	14	113
24,98	biozone C1	TGR-C	14	105
24,98	biozone C1	TGR-C	14	201
24,99	biozone C1	TGR-C	14	130
25,00	biozone C1	TGR-C	14	93
25,00	biozone C1	TGR-C	14	81
25,01	biozone C1	TGR-C	14	95
25,02	biozone C1	TGR-C	14	137
25,02	biozone C1	TGR-C	14	173
25,03	biozone C1	TGR-C	14	129
25,03	biozone C1	TGR-C	14	123
25,04	biozone C1	TGR-C	14	101
25,05	biozone C1	TGR-C	14	136
25,05	biozone C1	TGR-C	14	116
25,06	biozone C1	TGR-C	14	169
25,06	biozone C1	TGR-C	14	157
25,07	biozone C1	TGR-C	14	167
25,08	biozone C1	TGR-C	14	128
25,08	biozone C1	TGR-C	14	92
25,09	biozone C1	TGR-C	14	110
25,09	biozone C1	TGR-C	14	110
25,10	biozone C1	TGR-C	14	79
25,11	biozone C1	TGR-C	14	95
25,11	biozone C1	TGR-C	13	154
25,12	biozone C1	TGR-C	13	127
25,13	biozone C1	TGR-C	13	112
25,13	biozone C1	TGR-C	13	125
25,14	biozone C1	TGR-C	13	156
25,15	biozone C1	TGR-C	13	176
25,15	biozone C1	TGR-C	13	151
25,16	biozone C1	TGR-C	13	190
25,17	biozone C1	TGR-C	13	189
25,17	biozone C1	TGR-C	13	180
25,18	biozone C1	TGR-C	13	204
25,18	biozone C1	TGR-C	13	183
25,19	biozone C1	TGR-C	13	142
25,20	biozone C1	TGR-C	13	118
25,20	biozone C1	TGR-C	13	166
25,21	biozone C1	TGR-C	13	192
25,22	biozone C1	TGR-C	13	150
25,22	biozone C1	TGR-C	13	193
25,23	biozone C1	TGR-C	13	117
25,24	biozone C1	TGR-C	13	156
25,24	biozone C1	TGR-C	13	199
25,25	biozone C1	TGR-C	13	156
25,26	biozone C1	TGR-C	13	125
25,26	biozone C1	TGR-C	13	154
25,27	biozone C1	TGR-C	13	136
25,28	biozone C1	TGR-C	13	113
25,28	biozone C1	TGR-C	13	110
25,29	biozone C1	TGR-C	13	125
25,30	biozone C1	TGR-C	13	115
25,30	biozone C1	TGR-C	13	130
25,31	biozone C1	TGR-C	13	121
25,31	biozone C1	TGR-C	13	69
25,32	biozone C1	TGR-C	13	70
25,33	biozone C1	TGR-C	13	97
25,33	biozone C1	TGR-C	13	143

25,34	biozone C1	TGR-C	13	98
25,35	biozone C1	TGR-C	13	84
25,35	biozone C1	TGR-C	13	108
25,36	biozone C1	TGR-C	13	115
25,37	biozone C1	TGR-C	13	125
25,37	biozone C1	TGR-C	13	117
25,38	biozone C1	TGR-C	13	120
25,39	biozone C1	TGR-C	13	108
25,39	biozone C1	TGR-C	13	127
25,40	biozone C1	TGR-C	13	145
25,41	biozone C1	TGR-C	13	114
25,41	biozone C1	TGR-C	13	119
25,42	biozone C1	TGR-C	13	123
25,43	biozone C1	TGR-C	13	114
25,43	biozone C1	TGR-C	13	149
25,44	biozone C1	TGR-C	13	116
25,44	biozone C1	TGR-C	13	125
25,45	biozone C1	TGR-C	13	82
25,46	biozone C1	TGR-C	13	46
25,46	biozone C1	TGR-C	13	70
25,47	biozone C1	TGR-C	13	90
25,48	biozone C1	TGR-C	13	149
25,48	biozone C1	TGR-C	13	104
25,49	biozone C1	TGR-C	13	111
25,50	biozone C1	TGR-C	13	120
25,50	biozone C1	TGR-C	13	102
25,51	biozone C1	TGR-C	13	153
25,52	biozone C1	TGR-C	13	112
25,52	biozone C1	TGR-C	13	129
25,53	biozone C1	TGR-C	13	109
25,54	biozone C1	TGR-C	13	118
25,54	biozone C1	TGR-C	13	98
25,55	biozone C1	TGR-C	12	47
25,55	biozone C1	TGR-C	12	32
25,56	biozone C1	TGR-C	12	95
25,57	biozone C1	TGR-C	12	136
25,57	biozone C1	TGR-C	12	122
25,58	biozone C1	TGR-C	12	79
25,59	biozone C	TGR-C	11	58
25,59	biozone C	TGR-C	11	113
25,60	biozone C	TGR-C	11	83
25,61	biozone C	TGR-C	11	69
25,61	biozone C	TGR-C	11	94
25,62	biozone C	TGR-C	11	119
25,63	biozone C	TGR-C	11	91
25,63	biozone C	TGR-C	11	110
25,64	biozone C	TGR-C	11	115
25,65	biozone C	TGR-C	11	120
25,65	biozone C	TGR-C	11	115
25,66	biozone C	TGR-C	11	72
25,67	biozone C	TGR-C	11	137
25,67	biozone C	TGR-C	11	92
25,68	biozone C	TGR-C	11	91
25,68	biozone C	TGR-C	11	89
25,69	biozone C	TGR-C	11	97
25,70	biozone C	TGR-C	11	134
25,70	biozone C	TGR-C	11	107
25,71	biozone C	TGR-C	11	84
25,72	biozone C	TGR-C	11	133
25,72	biozone C	TGR-C	11	113
25,73	biozone C	TGR-C	11	103
25,74	biozone C	TGR-C	11	87
25,74	biozone C	TGR-C	11	131

25,75	biozone C	TGR-C	11	101
25,76	biozone C	TGR-C	11	116
25,76	biozone C	TGR-C	11	72
25,77	biozone C	TGR-C	11	68
25,78	biozone C	TGR-C	10	75
25,78	biozone C	TGR-C	10	70
25,79	biozone C	TGR-C	10	78
25,80	biozone C	TGR-C	10	106
25,80	biozone C	TGR-C	10	68
25,81	biozone C	TGR-C	10	69
25,81	biozone C	TGR-C	10	63
25,82	biozone C	TGR-C	10	42
25,83	biozone C	TGR-C	10	39
25,83	biozone C	TGR-C	10	36
25,84	biozone C	TGR-C	10	53
25,85	biozone C	TGR-C	10	54
25,85	biozone C	TGR-C	10	73
25,86	biozone C	TGR-C	10	42
25,87	biozone C	TGR-C	10	75
25,87	biozone C	TGR-C	10	81
25,88	biozone C	TGR-C	10	86
25,89	biozone C	TGR-C	10	126
25,89	biozone C	TGR-C	10	105
25,90	biozone C	TGR-C	10	73
25,91	biozone C	TGR-C	10	67
25,91	biozone C	TGR-C	10	120
25,92	biozone C	TGR-C	10	112
25,93	biozone C	TGR-C	10	81
25,93	biozone C	TGR-C	10	93
25,94	biozone C	TGR-C	10	58
25,94	biozone C	TGR-C	10	69
25,95	biozone C	TGR-C	10	59
25,96	biozone C	TGR-C	10	142
25,96	biozone C	TGR-C	10	96
25,97	biozone C	TGR-C	10	110
25,98	biozone C	TGR-C	10	114
25,98	biozone C	TGR-C	10	90
25,99	biozone C	TGR-C	10	101
26,00	biozone C	TGR-C	10	132
26,00	biozone C	TGR-C	10	175
26,01	biozone C	TGR-C	10	80
26,02	biozone C	TGR-C	10	117
26,02	biozone C	TGR-C	10	87
26,03	biozone C	TGR-C	9	130
26,04	biozone C	TGR-C	9	114
26,04	biozone C	TGR-C	9	96
26,05	biozone C	TGR-C	9	96
26,06	biozone C	TGR-C	9	90
26,06	biozone C	TGR-C	9	160
26,07	biozone C	TGR-C	9	144
26,07	biozone C	TGR-C	9	111
26,08	biozone C	TGR-C	9	89
26,09	biozone C	TGR-C	9	131
26,09	biozone C	TGR-C	9	109
26,10	biozone C	TGR-C	9	113
26,11	biozone C	TGR-C	9	141
26,11	biozone C	TGR-C	9	174
26,12	biozone C	TGR-C	8	119
26,13	biozone C	TGR-C	8	135
26,13	biozone C	TGR-C	8	140
26,14	biozone C	TGR-C	8	142
26,15	biozone C	TGR-C	8	156
26,15	biozone C	TGR-C	8	75

26,16	biozone C	TGR-C	8	120
26,17	biozone C	TGR-C	8	129
26,17	biozone C	TGR-C	8	144
26,18	biozone C	TGR-C	8	89
26,19	biozone C	TGR-C	8	97
26,19	biozone C	TGR-C	8	111
26,20	biozone C	TGR-C	8	149
26,20	biozone C	TGR-C	8	88
26,21	biozone C	TGR-C	8	58
26,22	biozone C	TGR-C	8	129
26,22	biozone C	TGR-C	8	136
26,23	biozone C	TGR-C	8	123
26,24	biozone C	TGR-C	8	143
26,24	biozone C	TGR-C	8	121
26,25	biozone C	TGR-C	8	145
26,26	biozone C	TGR-C	8	126
26,26	biozone C	TGR-C	8	188
26,27	biozone C	TGR-C	8	113
26,28	biozone C	TGR-C	8	139
26,28	biozone C	TGR-C	8	69
26,29	biozone C	TGR-C	8	162
26,30	biozone C	TGR-C	8	163
26,30	biozone C	TGR-C	8	92
26,31	biozone C	TGR-C	8	155
26,32	biozone C	TGR-C	8	170
26,32	biozone C	TGR-C	8	170
26,33	biozone C	TGR-C	8	145
26,33	biozone C	TGR-C	8	67
26,34	biozone C	TGR-C	8	112
26,35	biozone C	TGR-C	8	98
26,35	biozone C	TGR-C	8	54
26,36	biozone C	TGR-C	8	84
26,37	biozone C	TGR-C	7	82
26,37	biozone C	TGR-C	7	138
26,38	biozone C	TGR-C	7	146
26,39	biozone C	TGR-C	7	160
26,39	biozone C	TGR-C	7	193
26,40	biozone C	TGR-C	7	163
26,41	biozone C	TGR-C	7	160
26,41	biozone C	TGR-C	7	132
26,42	biozone C	TGR-C	7	123
26,43	biozone C	TGR-C	7	106
26,43	biozone C	TGR-C	7	119
26,44	biozone C	TGR-C	7	142
26,45	biozone C	TGR-C	7	72
26,45	biozone C	TGR-C	7	124
26,46	biozone C	TGR-C	7	119
26,46	biozone C	TGR-C	7	157
26,47	biozone C	TGR-C	7	163
26,48	biozone C	TGR-C	7	143
26,48	biozone C	TGR-C	7	107
26,49	biozone C	TGR-C	7	106
26,50	biozone C	TGR-C	7	144
26,50	biozone C	TGR-C	7	146
26,51	biozone C	TGR-C	7	200
26,52	biozone C	TGR-C	7	152
26,52	biozone C	TGR-C	7	138
26,53	biozone C	TGR-C	7	106
26,54	biozone C	TGR-C	7	177
26,54	biozone C	TGR-C	7	148
26,55	biozone C	TGR-C	7	121
26,56	biozone C	TGR-C	7	88
26,56	biozone C	TGR-C	7	128

26,57	biozone C	TGR-C	7	149
26,58	biozone C	TGR-C	7	139
26,58	biozone C	TGR-C	7	111
26,59	biozone C	TGR-C	7	121
26,59	biozone C	TGR-C	7	136
26,60	biozone C	TGR-C	7	178
26,61	biozone C	TGR-C	7	164
26,61	biozone C	TGR-C	6	106
26,62	biozone C	TGR-C	6	121
26,63	biozone C	TGR-C	6	127
26,63	biozone C	TGR-C	6	118
26,64	biozone C	TGR-C	6	70
26,65	biozone C	TGR-C	6	117
26,65	biozone C	TGR-C	6	59
26,66	biozone C	TGR-C	6	136
26,67	biozone C	TGR-C	6	78
26,67	biozone C	TGR-C	5	129
26,68	biozone C	TGR-C	5	124
26,69	biozone C	TGR-C	5	132
26,69	biozone C	TGR-C	5	144
26,70	biozone C	TGR-C	5	121
26,71	biozone C	TGR-C	5	124
26,71	biozone C	TGR-C	5	125
26,72	biozone C	TGR-C	5	125
26,72	biozone C	TGR-C	4	143
26,73	biozone C	TGR-C	4	109
26,74	biozone C	TGR-C	4	128
26,74	biozone C	TGR-C	4	186
26,75	biozone C	TGR-C	4	114
26,76	biozone C	TGR-C	4	111
26,76	biozone C	TGR-C	4	91
26,77	biozone C	TGR-C	4	118
26,78	biozone C	TGR-C	4	105
26,78	biozone C	TGR-C	4	115
26,79	biozone C	TGR-C	4	93
26,80	biozone C	TGR-C	4	117
26,80	biozone C	TGR-C	4	149
26,81	biozone C	TGR-C	4	168
26,82	biozone C	TGR-C	4	130
26,82	biozone C	TGR-C	4	143
26,83	biozone C	TGR-C	4	100
26,84	biozone C	TGR-C	4	168
26,84	biozone C	TGR-C	4	186
26,85	biozone C	TGR-C	4	167
26,85	biozone C	TGR-C	4	110
26,86	biozone C	TGR-C	4	113
26,87	biozone C	TGR-C	4	136
26,87	biozone C	TGR-C	4	131
26,88	biozone C	TGR-C	4	126
26,89	biozone C	TGR-C	4	129
26,89	biozone C	TGR-C	4	129
26,90	biozone C	TGR-C	4	119
26,91	biozone C	TGR-C	3A	34
26,91	biozone C	TGR-C	3A	83
26,92	biozone C	TGR-C	3A	65
26,93	biozone C	TGR-C	3A	61
26,93	biozone C	TGR-C	3A	71
26,94	biozone C	TGR-C	3A	87
26,95	biozone C	TGR-C	3	143
26,95	biozone C	TGR-C	3	138
26,96	biozone C	TGR-C	3	119
26,97	biozone C	TGR-C	3	129
26,97	biozone C	TGR-C	3	142

26,98	biozone C	TGR-C	3	126
26,98	biozone C	TGR-C	3	103
26,99	biozone C	TGR-C	3	151
27,00	biozone C	TGR-C	3	120
27,00	biozone C	TGR-C	3	116
27,01	biozone C	TGR-C	3	151
27,02	biozone C	TGR-C	3	143
27,02	biozone C	TGR-C	3	122
27,03	biozone C	TGR-C	3	134
27,04	biozone C	TGR-C	3	107
27,04	biozone C	TGR-C	3	142
27,05	biozone C	TGR-C	3	116
27,06	biozone C	TGR-C	3	94
27,06	biozone C	TGR-C	3	113
27,07	biozone C	TGR-C	3	84
27,08	biozone C	TGR-C	3	115
27,08	biozone C	TGR-C	3	118
27,09	biozone C	TGR-C	3	105
27,10	biozone C	TGR-C	3	88
27,10	biozone C	TGR-C	3	126
27,11	biozone C	TGR-C	3	124
27,11	biozone C	TGR-C	3	125
27,12	biozone C	TGR-C	3	92
27,13	biozone C	TGR-C	3	114
27,13	biozone C	TGR-C	3	131
27,14	biozone C	TGR-C	3	87
27,15	biozone C	TGR-C	3	80
27,15	biozone C	TGR-C	3	82
27,16	biozone C	TGR-C	3	136
27,17	biozone C	TGR-C	3	93
27,17	biozone C	TGR-C	3	106
27,18	biozone C	TGR-C	3	102
27,19	biozone C	TGR-C	3	135
27,19	biozone C	TGR-C	3	130
27,20	biozone C	TGR-C	3	131
27,21	biozone C	TGR-C	3	107
27,21	biozone C	TGR-C	3	114
27,22	biozone C	TGR-C	3	105
27,23	biozone C	TGR-C	3	129
27,23	biozone C	TGR-C	3	101
27,24	biozone C	TGR-C	3	129
27,24	biozone C	TGR-C	3	114
27,25	biozone C	TGR-C	3	127
27,26	biozone C	TGR-C	3	126
27,26	biozone C	TGR-C	3	161
27,27	biozone C	TGR-C	3	135
27,28	biozone C	TGR-C	3	93
27,28	biozone C	TGR-C	3	129
27,29	biozone C	TGR-C	3	76
27,30	biozone C	TGR-C	3	89
27,30	biozone C	TGR-C	3	69
27,31	biozone C	TGR-C	3	130
27,32	biozone C	TGR-C	3	78
27,32	biozone C	TGR-C	3	102
27,33	biozone C	TGR-C	2	101
27,34	biozone C	TGR-C	2	101
27,34	biozone C	TGR-C	2	46
27,35	biozone C	TGR-C	2	95
27,36	biozone C	TGR-C	2	89
27,36	biozone C	TGR-C	2	92
27,37	biozone C	TGR-C	2	137
27,37	biozone C	TGR-C	2	119
27,38	biozone C	TGR-C	2	129

27,39	biozone C	TGR-C	2	134
27,39	biozone C	TGR-C	2	118
27,40	biozone C	TGR-C	2	166
27,41	biozone C	TGR-C	1	155
27,41	biozone C	TGR-C	1	80
27,42	biozone C	TGR-C	1	130
27,43	biozone C	TGR-C	1	93
27,43	biozone C	TGR-C	1	94
27,44	biozone C	TGR-C	1	141
28,00	biozone B	SHG	26	92
28,01	biozone B	SHG	26	135
28,01	biozone B	SHG	26	47
28,02	biozone B	SHG	26	90
28,03	biozone B	SHG	26	84
28,04	biozone B	SHG	26	101
28,04	biozone B	SHG	26	93
28,05	biozone B	SHG	26	150
28,06	biozone B	SHG	26	135
28,07	biozone B	SHG	26	190
28,07	biozone B	SHG	26	144
28,08	biozone B	SHG	26	98
28,09	biozone B	SHG	26	122
28,09	biozone B	SHG	26	111
28,10	biozone B	SHG	26	176
28,11	biozone B	SHG	26	151
28,12	biozone B	SHG	26	128
28,12	biozone B	SHG	26	142
28,13	biozone B	SHG	26	92
28,14	biozone B	SHG	25	106
28,15	biozone B	SHG	25	97
28,15	biozone B	SHG	25	100
28,16	biozone B	SHG	25	131
28,17	biozone B	SHG	25	128
28,18	biozone B	SHG	25	121
28,18	biozone B	SHG	25	110
28,19	biozone B	SHG	25	137
28,20	biozone B	SHG	25	165
28,20	biozone B	SHG	24	116
28,21	biozone B	SHG	24	100
28,22	biozone B	SHG	24	65
28,23	biozone B	SHG	24	116
28,23	biozone B	SHG	24	70
28,24	biozone B	SHG	24	113
28,25	biozone B	SHG	24	123
28,26	biozone B	SHG	23	76
28,26	biozone B	SHG	23	109
28,27	biozone B	SHG	23	119
28,28	biozone B	SHG	23	119
28,28	biozone B	SHG	23	80
28,29	biozone B	SHG	23	101
28,30	biozone B	SHG	23	94
28,31	biozone B	SHG	23	109
28,31	biozone B	SHG	23	95
28,32	biozone B	SHG	23	81
28,33	biozone B	SHG	23	100
28,34	biozone B	SHG	23	64
28,34	biozone B	SHG	23	62
28,35	biozone B	SHG	23	68
28,36	biozone B	SHG	23	74
28,37	biozone B	SHG	23	91
28,37	biozone B	SHG	23	50
28,38	biozone B	SHG	23	100
28,39	biozone B	SHG	23	86

28,39	biozone B	SHG	23	68
28,40	biozone B	SHG	23	89
28,41	biozone B	SHG	23	81
28,42	biozone B	SHG	23	101
28,42	biozone B	SHG	23	103
28,43	biozone B	SHG	23	89
28,44	biozone B	SHG	23	43
28,45	biozone B	SHG	23	98
28,45	biozone B	SHG	23	90
28,46	biozone B	SHG	23	92
28,47	biozone B	SHG	23	77
28,47	biozone B	SHG	23	105
28,48	biozone B	SHG	23	95
28,49	biozone B	SHG	23	96
28,50	biozone B	SHG	23	90
28,50	biozone B	SHG	23	95
28,51	biozone B	SHG	23	68
28,52	biozone B	SHG	23	123
28,53	biozone B	SHG	23	120
28,53	biozone B	SHG	23	127
28,54	biozone B	SHG	23	107
28,55	biozone B	SHG	23	86
28,55	biozone B	SHG	23	95
28,56	biozone B	SHG	23	153
28,57	biozone B	SHG	23	106
28,58	biozone B	SHG	23	79
28,58	biozone B	SHG	23	82
28,59	biozone B	SHG	23	104
28,60	biozone B	SHG	23	105
28,61	biozone B	SHG	23	115
28,61	biozone B	SHG	23	84
28,62	biozone B	SHG	23	113
28,63	biozone B	SHG	23	83
28,64	biozone B	SHG	23	61
28,64	biozone B	SHG	23	115
28,65	biozone B	SHG	23	135
28,66	biozone B	SHG	23	87
28,66	biozone B	SHG	23	64
28,67	biozone B	SHG	23	51
28,68	biozone B	SHG	23	82
28,69	biozone B	SHG	23	90
28,69	biozone B	SHG	23	92
28,70	biozone B	SHG	23	72
28,71	biozone B	SHG	23	98
28,72	biozone B	SHG	23	89
28,72	biozone B	SHG	23	107
28,73	biozone B	SHG	23	97
28,74	biozone B	SHG	23	111
28,74	biozone B	SHG	23	116
28,75	biozone B	SHG	23	120
28,76	biozone B	SHG	23	69
28,77	biozone B	SHG	23	82
28,77	biozone B	SHG	23	59
28,78	biozone B	SHG	23	136
28,79	biozone B	SHG	23	114
28,80	biozone B	SHG	23	96
28,80	biozone B	SHG	23	109
28,81	biozone B	SHG	23	140
28,82	biozone B	SHG	23	83
28,82	biozone B	SHG	23	74
28,83	biozone B	SHG	23	111
28,84	biozone B	SHG	23	175
28,85	biozone B	SHG	23	118

28,85	biozone B	SHG	22	113
28,86	biozone B	SHG	22	131
28,87	biozone B	SHG	21	105
28,88	biozone B	SHG	21	208
28,88	biozone B	SHG	21	160
28,89	biozone B	SHG	21	139
28,90	biozone B	SHG	21	150
28,91	biozone B	SHG	21	137
28,91	biozone B	SHG	21	154
28,92	biozone B	SHG	21	181
28,93	biozone B	SHG	21	126
28,93	biozone B	SHG	21	245
28,94	biozone B	SHG	21	157
28,95	biozone B	SHG	21	177
28,96	biozone B	SHG	20	116
28,96	biozone B	SHG	20	128
28,97	biozone B	SHG	20	135
28,98	biozone B	SHG	20	63
28,99	biozone B	SHG	20	104
28,99	biozone B	SHG	20	82
29,00	biozone B	SHG	20	155
29,01	biozone B	SHG	20	99
29,01	biozone B	SHG	20	73
29,02	biozone B	SHG	20	89
29,03	biozone B	SHG	20	133
29,04	biozone B	SHG	20	101
29,04	biozone B	SHG	20	116
29,05	biozone B	SHG	20	92
29,06	biozone B	SHG	20	85
29,07	biozone B	SHG	19	66
29,07	biozone B	SHG	19	144
29,08	biozone B	SHG	19	130
29,09	biozone B	SHG	19	117
29,10	biozone B	SHG	19	157
29,10	biozone B	SHG	19	170
29,11	biozone B	SHG	19	156
29,12	biozone B	SHG	19	144
29,12	biozone B	SHG	19	121
29,13	biozone B	SHG	19	166
29,14	biozone B	SHG	18	148
29,15	biozone B	SHG	18	167
29,15	biozone B	SHG	18	121
29,16	biozone B	SHG	18	126
29,17	biozone B	SHG	17	154
29,18	biozone B	SHG	17	185
29,18	biozone B	SHG	17	143
29,19	biozone B	SHG	17	161
29,20	biozone B	SHG	17	90
29,20	biozone B	SHG	17	184
29,21	biozone B	SHG	17	127
29,22	biozone B	SHG	16	126
29,23	biozone B	SHG	16	168
29,23	biozone B	SHG	16	165
29,24	biozone B	SHG	16	97
29,25	biozone B	SHG	16	134
29,26	biozone B	SHG	16	122
29,26	biozone B	SHG	16	129
29,27	biozone B	SHG	16	87
29,28	biozone B	SHG	16	136
29,28	biozone B	SHG	16	94
29,29	biozone B	SHG	16	76
29,30	biozone B	SHG	16	110
29,31	biozone B	SHG	16	137

29,31	biozone B	SHG	16	75
29,32	biozone B	SHG	16	86
29,33	biozone B	SHG	16	108
29,34	biozone B	SHG	16	124
29,34	biozone B	SHG	16	127
29,35	biozone B	SHG	16	113
29,36	biozone B	SHG	16	160
29,37	biozone B	SHG	16	152
29,37	biozone B	SHG	16	109
29,38	biozone B	SHG	15	152
29,39	biozone B	SHG	15	148
29,39	biozone B	SHG	15	124
29,40	biozone B	SHG	15	139
29,41	biozone B	SHG	15	164
29,42	biozone B	SHG	15	183
29,42	biozone B	SHG	15	183
29,43	biozone B	SHG	15	170
29,44	biozone B	SHG	15	154
29,45	biozone B	SHG	15	173
29,45	biozone B	SHG	15	120
29,46	biozone B	SHG	14	82
29,47	biozone B	SHG	14	75
29,47	biozone B	SHG	14	133
29,48	biozone B	SHG	14	92
29,49	biozone B	SHG	14	100
29,50	biozone B	SHG	14	100
29,50	biozone B	SHG	14	101
29,51	biozone B	SHG	14	123
29,52	biozone B	SHG	14	91
29,53	biozone B	SHG	14	96
29,53	biozone B	SHG	14	84
29,54	biozone B	SHG	14	83
29,55	biozone B	SHG	13	124
29,55	biozone B	SHG	13	109
29,56	biozone B	SHG	13	154
29,57	biozone B	SHG	13	143
29,58	biozone B	SHG	13	99
29,58	biozone B	SHG	13	113
29,59	biozone B	SHG	13	95
29,60	biozone B	SHG	12	47
29,61	biozone B	SHG	12	77
29,61	biozone B	SHG	12	88
29,62	biozone B	SHG	12	78
29,63	biozone B	SHG	12	25
29,64	biozone B	SHG	12	27
29,64	biozone B	SHG	12	57
29,65	biozone B	SHG	12	66
29,66	biozone B	SHG	12	88
29,66	biozone B	SHG	12	89
29,67	biozone B	SHG	12	93
29,68	biozone B	SHG	12	77
29,69	biozone B	SHG	12	91
29,69	biozone B	SHG	12	110
29,70	biozone B	SHG	12	102
29,71	biozone B	SHG	12	105
29,72	biozone B	SHG	12	67
29,72	biozone B	SHG	12	79
29,73	biozone B	SHG	12	96
29,74	biozone B	SHG	12	81
29,74	biozone B	SHG	12	67
29,75	biozone B	SHG	12	110
29,76	biozone B	SHG	12	118
29,77	biozone B	SHG	12	95

29,77	biozone B	SHG	12	89
29,78	biozone B	SHG	12	36
29,79	biozone B	SHG	12	61
29,80	biozone B	SHG	12	97
29,80	biozone B	SHG	12	106
29,81	biozone B	SHG	12	115
29,82	biozone B	SHG	11	74
29,83	biozone B	SHG	11	94
29,83	biozone B	SHG	11	82
29,84	biozone B	SHG	11	72
29,85	biozone B	SHG	11	143
29,85	biozone B	SHG	11	138
29,86	biozone B	SHG	11	134
29,87	biozone B	SHG	11	104
29,88	biozone B	SHG	11	163
29,88	biozone B	SHG	11	145
29,89	biozone B	SHG	11	144
29,90	biozone B	SHG	11	122
29,91	biozone B	SHG	11	144
29,91	biozone B	SHG	11	159
29,92	biozone B	SHG	11	94
29,93	biozone B	SHG	11	98
29,93	biozone B	SHG	11	122
29,94	biozone B	SHG	11	101
29,95	biozone B	SHG	11	133
29,96	biozone B	SHG	11	113
29,96	biozone B	SHG	11	95
29,97	biozone B	SHG	11	100
29,98	biozone B	SHG	11	151
29,99	biozone B	SHG	11	103
29,99	biozone B	SHG	11	175
30,00	biozone B	SHG	11	88
30,01	biozone B	SHG	11	122
30,01	biozone B	SHG	11	161
30,02	biozone B	SHG	11	125
30,03	biozone B	SHG	11	137
30,04	biozone B	SHG	11	117
30,04	biozone B	SHG	11	174
30,05	biozone B	SHG	11	168
30,06	biozone B	SHG	11	150
30,07	biozone B	SHG	11	110
30,07	biozone B	SHG	11	105
30,08	biozone B	SHG	11	117
30,09	biozone B	SHG	11	157
30,10	biozone B	SHG	11	164
30,10	biozone B	SHG	11	102
30,11	biozone B	SHG	11	152
30,12	biozone B	SHG	11	126
30,12	biozone B	SHG	11	120
30,13	biozone B	SHG	11	136
30,14	biozone B	SHG	11	142
30,15	biozone B	SHG	11	133
30,15	biozone B	SHG	11	107
30,16	biozone B	SHG	10	128
30,17	biozone B	SHG	10	136
30,18	biozone B	SHG	10	66
30,18	biozone B	SHG	9	112
30,19	biozone B	SHG	9	145
30,20	biozone B	SHG	9	158
30,20	biozone B	SHG	9	166
30,21	biozone B	SHG	9	158
30,22	biozone B	SHG	9	157
30,23	biozone B	SHG	9	150

30,23	biozone B	SHG	9	206
30,24	biozone B	SHG	9	157
30,25	biozone B	SHG	9	159
30,26	biozone B	SHG	9	112
30,26	biozone B	SHG	9	132
30,27	biozone B	SHG	9	154
30,28	biozone B	SHG	9	121
30,28	biozone B	SHG	9	144
30,29	biozone B	SHG	9	175
30,30	biozone B	SHG	9	127
30,31	biozone B	SHG	9	146
30,31	biozone B	SHG	9	104
30,32	biozone B	SHG	9	79
30,33	biozone B	SHG	9	98
30,34	biozone B	SHG	8	95
30,34	biozone B	SHG	8	132
30,35	biozone B	SHG	8	112
30,36	biozone B	SHG	7	135
30,37	biozone B	SHG	7	180
30,37	biozone B	SHG	7	173
30,38	biozone B	SHG	7	186
30,39	biozone B	SHG	7	157
30,39	biozone B	SHG	7	118
30,40	biozone B	SHG	7	159
30,41	biozone B	SHG	7	211
30,42	biozone B	SHG	7	141
30,42	biozone B	SHG	7	179
30,43	biozone B	SHG	7	124
30,44	biozone B	SHG	7	146
30,45	biozone B	SHG	7	199
30,45	biozone B	SHG	7	192
30,46	biozone B	SHG	7	111
30,47	biozone B	SHG	7	133
30,47	biozone B	SHG	7	102
30,48	biozone B	SHG	7	150
30,49	biozone B	SHG	7	115
30,50	biozone B	SHG	7	124
30,50	biozone B	SHG	7	112
30,51	biozone B	SHG	7	144
30,52	biozone B	SHG	7	134
30,53	biozone B	SHG	7	123
30,53	biozone B	SHG	7	166
30,54	biozone B	SHG	7	162
30,55	biozone B	SHG	7	162
30,56	biozone B	SHG	7	150
30,56	biozone B	SHG	7	166
30,57	biozone B	SHG	7	150
30,58	biozone B	SHG	7	163
30,58	biozone B	SHG	7	103
30,59	biozone B	SHG	7	127
30,60	biozone B	SHG	7	131
30,61	biozone B	SHG	7	125
30,61	biozone B	SHG	7	168
30,62	biozone B	SHG	7	105
30,63	biozone B	SHG	7	126
30,64	biozone B	SHG	7	110
30,64	biozone B	SHG	7	106
30,65	biozone B	SHG	7	183
30,66	biozone B	SHG	7	138
30,66	biozone B	SHG	7	138
30,67	biozone B	SHG	7	130
30,68	biozone B	SHG	7	151
30,69	biozone B	SHG	6	93

30,69	biozone B	SHG	6	117
30,70	biozone B	SHG	6	130
30,71	biozone B	SHG	6	111
30,72	biozone B	SHG	6	135
30,72	biozone B	SHG	5	100
30,73	biozone B	SHG	5	123
30,74	biozone B	SHG	5	133
30,74	biozone B	SHG	5	153
30,75	biozone B	SHG	5	117
30,76	biozone B	SHG	5	103
30,77	biozone B	SHG	5	129
30,77	biozone B	SHG	5	152
30,78	biozone B	SHG	4	139
30,79	biozone B	SHG	4	110
30,80	biozone B	SHG	4	162
30,80	biozone B	SHG	4	58
30,81	biozone B	SHG	4	45
30,82	biozone B	SHG	4	158
30,83	biozone B	SHG	4	172
30,83	biozone B	SHG	4	172
30,84	biozone B	SHG	4	163
30,85	biozone B	SHG	3	137
30,85	biozone B	SHG	3	174
30,86	biozone B	SHG	3	190
30,87	biozone B	SHG	3	161
30,88	biozone B	SHG	3	121
30,88	biozone B	SHG	3	137
30,89	biozone B	SHG	3	170
30,90	biozone B	SHG	3	131
30,91	biozone B	SHG	3	170
30,91	biozone B	SHG	3	183
30,92	biozone B	SHG	3	152
30,93	biozone B	SHG	3	143
30,93	biozone B	SHG	3	176
30,94	biozone B	SHG	3	144
30,95	biozone B	SHG	3	89
30,96	biozone B	SHG	3	171
30,96	biozone B	SHG	3	133
30,97	biozone B	SHG	3	162
30,98	biozone B	SHG	3	101
30,99	biozone B	SHG	3	130
30,99	biozone B	SHG	3	148
31,00	biozone B	SHG	3	136
31,01	biozone B	SHG	3	92
31,01	biozone B	SHG	3	152
31,02	biozone B	SHG	3	142
31,03	biozone B	SHG	3	114
31,04	biozone B	SHG	3	146
31,04	biozone B	SHG	2	65
31,05	biozone B	SHG	2	146
31,06	biozone B	SHG	2	75
31,07	biozone B	SHG	2	79
31,07	biozone B	SHG	2	91
31,08	biozone B	SHG	2	164
31,09	biozone B	SHG	2	103
31,10	biozone B	SHG	2	141
31,10	biozone B	SHG	2	87
31,11	biozone B	SHG	2	88
31,12	biozone B	SHG	2	128
31,12	biozone B	SHG	2	75
31,13	biozone B	SHG	2	63
31,14	biozone B	SHG	2	130
31,15	biozone B	SHG	2	79

31,15	biozone B	SHG	2	126
31,16	biozone B	SHG	2	48
31,17	biozone B	SHG	1	72
31,18	biozone B	SHG	1	222
31,18	biozone B	SHG	1	165
31,19	biozone B	SHG	1	155
31,20	biozone B	SHG	1	208
31,20	biozone B	SHG	1	200
31,21	biozone B	SHG	1	178
31,22	biozone B	SHG	1	215
31,23	biozone B	SHG	1	134
31,23	biozone B	SHG	1	179
31,24	biozone B	SHG	1	158
31,25	biozone B	SHG	1	126
31,26	biozone B	SHG	1	60
31,26	biozone B	SHG	1	113
31,27	biozone B	SHG	1	196
31,28	biozone B	SHG	1	142
31,29	biozone B	SHG	1	157
31,29	biozone B	SHG	1	111
31,30	biozone B	SHG	1	132
31,31	biozone B	SHG	1	164
31,31	biozone B	SHG	1	211
31,32	biozone B	SHG	1	223
31,33	biozone B	SHG	1	208
31,34	biozone B	SHG	1	165
31,34	biozone B	SHG	1	172
31,35	biozone B	SHG	1	168
31,36	biozone B	SHG	1	205
31,37	biozone B	SHG	1	148
31,37	biozone B	SHG	1	199
31,38	biozone B	SHG	1	197
31,39	biozone B	SHG	1	119
31,39	biozone B	SHG	1	131
31,40	biozone B	SHG	1	161
31,41	biozone B	SHG	1	136
32,00	biozone A	TGR	32	211
32,02	biozone A	TGR	32	227
32,03	biozone A	TGR	32	249
32,05	biozone A	TGR	32	245
32,07	biozone A	TGR	32	158
32,09	biozone A	TGR	32	239
32,10	biozone A	TGR	32	153
32,12	biozone A	TGR	32	226
32,14	biozone A	TGR	32	220
32,16	biozone A	TGR	32	165
32,17	biozone A	TGR	32	180
32,19	biozone A	TGR	32	166
32,21	biozone A	TGR	32	183
32,23	biozone A	TGR	32	233
32,24	biozone A	TGR	32	149
32,26	biozone A	TGR	32	206
32,28	biozone A	TGR	32	170
32,30	biozone A	TGR	32	112
32,31	biozone A	TGR	32	200
32,33	biozone A	TGR	32	203
32,35	biozone A	TGR	32	156
32,37	biozone A	TGR	32	129
32,38	biozone A	TGR	32	114
32,40	biozone A	TGR	32	154
32,42	biozone A	TGR	32	156
32,44	biozone A	TGR	32	156
32,45	biozone A	TGR	32	176

32,47	biozone A	TGR	32	219
32,49	biozone A	TGR	32	229
32,50	biozone A	TGR	32	218
32,52	biozone A	TGR	32	259
32,54	biozone A	TGR	32	295
32,56	biozone A	TGR	32	275
32,57	biozone A	TGR	32	257
32,59	biozone A	TGR	32	158
32,61	biozone A	TGR	32	168
32,63	biozone A	TGR	32	185
32,64	biozone A	TGR	32	160
32,66	biozone A	TGR	32	156
32,68	biozone A	TGR	32	187
32,70	biozone A	TGR	32	236
32,71	biozone A	TGR	32	239
32,73	biozone A	TGR	32	139
32,75	biozone A	TGR	32	162
32,77	biozone A	TGR	32	194
32,78	biozone A	TGR	32	140
32,80	biozone A	TGR	32	214
32,82	biozone A	TGR	32	186
32,84	biozone A	TGR	32	251
32,85	biozone A	TGR	32	245
32,87	biozone A	TGR	32	148
32,89	biozone A	TGR	32	198
32,90	biozone A	TGR	32	191
32,92	biozone A	TGR	32	228
32,94	biozone A	TGR	32	200
32,96	biozone A	TGR	32	196
32,97	biozone A	TGR	32	131
32,99	biozone A	TGR	32	155
33,01	biozone A	TGR	32	125
33,03	biozone A	TGR	32	133
33,04	biozone A	TGR	32	153
33,06	biozone A	TGR	32	201
33,08	biozone A	TGR	32	220
33,10	biozone A	TGR	32	194
33,11	biozone A	TGR	32	149
33,13	biozone A	TGR	32	215
33,15	biozone A	TGR	32	205
33,17	biozone A	TGR	32	149
33,18	biozone A	TGR	32	171
33,20	biozone A	TGR	32	217
33,22	biozone A	TGR	32	150
33,24	biozone A	TGR	32	243
33,25	biozone A	TGR	32	269
33,27	biozone A	TGR	32	191
33,29	biozone A	TGR	32	224
33,31	biozone A	TGR	32	246
33,32	biozone A	TGR	32	116
33,34	biozone A	TGR	32	139
33,36	biozone A	TGR	32	231
33,37	biozone A	TGR	32	194
33,39	biozone A	TGR	32	116
33,41	biozone A	TGR	32	153
33,43	biozone A	TGR	32	155
33,44	biozone A	TGR	32	140
33,46	biozone A	TGR	32	182
33,48	biozone A	TGR	32	180
33,50	biozone A	TGR	32	100
33,51	biozone A	TGR	32	133
33,53	biozone A	TGR	32	161
33,55	biozone A	TGR	32	97

33,57	biozone A	TGR	32	120
33,58	biozone A	TGR	32	144
33,60	biozone A	TGR	32	124
33,62	biozone A	TGR	32	156
33,64	biozone A	TGR	32	191
33,65	biozone A	TGR	31	186
33,67	biozone A	TGR	31	194
33,69	biozone A	TGR	31	189
33,71	biozone A	TGR	31	208
33,72	biozone A	TGR	31	146
33,74	biozone A	TGR	31	205
33,76	biozone A	TGR	31	170
33,77	biozone A	TGR	31	141
33,79	biozone A	TGR	31	140
33,81	biozone A	TGR	29a-30	175
33,83	biozone A	TGR	29a-30	122
33,84	biozone A	TGR	29a-30	135
33,86	biozone A	TGR	29a-30	106
33,90	biozone A	TGR	29a-30	158
33,91	biozone A	TGR	29a-30	106
33,92	Eocene	TGR	28a	46
33,93	Eocene	TGR	28a	70
33,94	Eocene	TGR	28a	99
33,95	Eocene	TGR	28a	50
33,96	Eocene	TGR	28a	66
33,97	Eocene	TGR	28a	80
